# Assessment of *Annona muricata* Phytochemicals as 17β‐HSD1 Inhibitors through Molecular Docking, Dynamics Simulation, DFT, and ADMET Analyses

**DOI:** 10.1111/jcmm.70904

**Published:** 2025-11-12

**Authors:** Emad Rashad Sindi, Md Jannatul Islam Polash, Guilherme Bastos Alves, Imren Bayil, Samson Olusegun Afolabi, Hanan M. Alharbi, Alaa A. Khojah, Jonas Ivan Nobre Oliveira, Aamal A. Al‐Mutairi, Magdi E. A. Zaki

**Affiliations:** ^1^ Division of Clinical Biochemistry, Department of Basic Medical Sciences, College of Medicine University of Jeddah Jeddah Saudi Arabia; ^2^ Department of Pharmacy Islamic University Bangladesh; ^3^ Department of Biophysics and Pharmacology, Bioscience Center Federal University of Rio Grande Do Norte Natal Brazil; ^4^ Department of Bioinformatics and Computational Biology Gaziantep University Turkey; ^5^ Department of Chemistry Federal University of Agriculture Abeokuta Nigeria; ^6^ Department of Pharmaceutical Sciences, College of Pharmacy Umm Al‐Qura University Makkah Saudi Arabia; ^7^ Department of Chemistry, College of Science Imam Mohammad Ibn Saud Islamic University (IMSIU) Riyadh Kingdom of Saudi Arabia

**Keywords:** ADMET, *Annona muricata*, breast cancer, molecular docking, molecular dynamic simulation

## Abstract

Breast cancer remains a leading cause of female mortality, largely sustained by local oestrogen production. The enzyme 17β‐hydroxysteroid dehydrogenase type 1 (17β‐HSD1) is an enzyme involved in the oestrogen biosynthesis pathway, particularly in the conversion of estrone to estradiol, and is overexpressed in conditions such as breast cancer. Therefore, it is considered a relevant target for drug development. We evaluated phytocompounds from *Annona muricata* as prospective 17β‐HSD1 inhibitors through an integrated in silico workflow. Twelve reported constituents (myristic acid, myrcene, palmitic acid, hexanoic acid, pentadecane, methyl 3‐phenylpropionate, butyric acid, linalool, reticuline, phytol, camphene and calamenene) were geometry‐optimised by density‐functional theory, docked against 17β‐HSD1 (PDB 3HB5), and screened for drug‐likeness and ADMET liabilities. The top‐ranked complexes—reticuline (ΔG_pred = −8.4 kcal mol^−1^) and calamenene (−7.7 kcal mol^−1^)—scored more favourably than the reference epirubicin (−5.7 kcal mol^−1^) and were subjected to 100 ns molecular‐dynamics simulations. Both ligands remained stably anchored within the catalytic pocket, with root mean square deviation (RMSD) fluctuations below 2.0 Å, yet MM‐PBSA binding free energies (reticuline −27 kJ mol^−1^; calamenene −9 kJ mol^−1^) did not surpass the reference. ADMET profiling predicted acceptable Caco‐2 permeability (0.92–0.94), and only moderate hERG liability, but also signalled potential DILI and carcinogenicity alerts that warrant caution. Reticuline and calamenene fulfilled all of Lipinski's criteria, with the exception of logP in the case of calamenene. Collectively, these findings nominate reticuline and calamenene as promising lead scaffolds for selective intracellular suppression of oestrogen biosynthesis. Experimental validation (enzyme kinetics, cell‐based assays and pharmacokinetic studies) is warranted to confirm their clinical potential in oestrogen‐dependent breast cancer.

**Abbreviations**
17β‐HSD1: 17β‐Hydroxysteroid Dehydrogenase Type 1
ADMET: absorption, distribution, metabolism, excretion and toxicity
ADT: AutoDock tools
ALA: Alanine
ARG: Arginine
BBB: blood–brain barrier
CHARMM: chemistry at harvard macromolecular mechanics
CYP450: Cytochrome P450
DCCM: dynamic cross‐correlation matrix
DFT: density functional theory
DILI: drug‐induced liver injury
EEEL (ΔEEEL): electrostatic energy (MM‐PBSA term)
EVDW (ΔEVDW): van der Waals energy (MM‐PBSA term)
GLY: Glycine
HADDOCK: high ambiguity driven DOCKing
hERG: human Ether‐à‐go‐go Related Gene
HOMO: highest occupied molecular orbital
LINCS: linear constraint solver
LUMO: lowest unoccupied molecular orbital
MD: molecular dynamics
MM‐PBSA: molecular mechanics poisson–boltzmann surface area
MW: molecular weight
NPT: constant number, pressure and temperature ensemble
NVT: constant number, volume and temperature ensemble
PCA: principal component analysis
PDB: protein data bank
Pgp: P‐glycoprotein
PK: pharmacokinetics
PME: particle mesh EWALD
PPB: plasma protein binding
Rg: radius of gyration
RMSD: root mean square deviation
RMSF: root mean square fluctuation
VMD: visual molecular dynamics

## 1 Introduction

Breast cancer, which accounted for 11.6% of cancers diagnosed in 2022, remains a major global health problem owing to its complex pathophysiology, heterogeneous clinical presentation and the persistent challenges in developing effective therapeutic strategies [1]. Lifetime risk estimates show that in very‐high Human Development Index (HDI) countries, one in twelve women will develop the disease and one in seventy‐one will die from it, whereas in low‐HDI settings the ratios are approximately 1:27 and 1:48, respectively [2].

Nearly 70% of breast tumours are driven by oestrogens and are therefore classified as hormone‐dependent [3, 4]. The potent oestrogen 17β‐estradiol (E2) fuels the initiation and progression of hormone‐dependent breast cancers (HDBCs) [5, 6]. Consequently, attenuating oestrogen signalling remains a major therapeutic strategy. Current approaches focus on blocking oestrogen receptor‐α with selective oestrogen‐receptor modulators or related anti‐oestrogens [7, 8]; however, resistance frequently emerges [9], underscoring the need for alternative interventions that deplete intracellular oestrogen levels directly.

Oestrogen biosynthesis relies on several enzymes [10]; among them, steroid sulfatase (STS), aromatase and 17β‐hydroxysteroid dehydrogenase type 1 (17β‐HSD1) are the most clinically relevant [11, 12]. Thus far, only aromatase has yielded marketed drugs—letrozole, exemestane and anastrozole—for breast cancer therapy [13]. STS inhibitors are less advanced: irosustat is the sole compound to reach phase II trials, and larger studies are still required [13, 14, 15]. Unfortunately, systemic suppression of oestrogen activity is often accompanied by severe adverse events such as stroke, thrombosis, osteoporosis and endometrial cancer [16, 17, 18]. A strategy that selectively blocks intracellular E2 synthesis in tumour tissue would therefore be highly advantageous.

17β‐HSD1 is intimately involved in oestrogen production and tumour proliferation [3, 19]. Besides converting estrone into E2, it catalyses the transformation of DHEA into 5‐androstene‐3β,17β‐diol (5‐diol), whose levels rise after menopause [20]. Overexpression of 17β‐HSD1 in breast cancer cells accelerates the NADPH‐dependent conversion of estrone (E1) to E2 [18, 21], making selective inhibition of this final biosynthetic step an appealing therapeutic approach.

Despite intensive efforts since the early 2000s, no 17β‐HSD1 inhibitor has yet entered clinical use for breast cancer [22]. The most advanced candidates have been evaluated only in phase I studies for endometriosis [23]. Many early inhibitors, such as 16β‐(m‐carbamoylbenzyl)‐E2, showed high biochemical potency but paradoxically stimulated proliferation in ER‐positive T‐47D and MCF‐7 cells, limiting their translational value [3, 24]. Validation of 17β‐HSD1 blockade as a viable anticancer strategy, therefore, remains elusive.

In recent years, natural‐product‐derived substances have emerged as promising alternatives in the discovery of novel therapeutic agents, either as isolated compounds or semisynthetic derivatives [25]. *Annona muricata* (soursop or graviola) exhibits diverse pharmacological activities, including pronounced cytotoxicity against breast cancer cells [26, 27]. Here, we investigate twelve *A. muricata* phytochemicals (myristic acid, myrcene, palmitic acid, hexanoic acid, pentadecane, methyl 3‐phenylpropionate, butyric acid, linalool, reticuline, phytol, camphene and calamenene), selected as representative and biologically relevant constituents reflecting the chemical diversity and pharmacological potential of the species, as putative 17β‐HSD1 inhibitors using density‐functional theory (DFT), molecular docking, molecular‐dynamics simulations, and ADMET profiling. This integrative workflow aims to identify safe and effective lead compounds for the future development of targeted therapies against oestrogen‐dependent breast cancer, providing a predictive basis for subsequent experimental validation.

## 2 Working Methods

### 2.1 Quantum Analysis

The most stable conformers were chosen for further quantum‐chemical calculations using the Gaussian 16 Rev. C.01 software (https://gaussian.com/). Geometry optimizations and quantum‐mechanical calculations were performed with density‐functional theory (DFT) employing the B3LYP hybrid exchange–correlation functional and the aug‐cc‐pVTZ (augmented correlation‐consistent polarized valence triple‐zeta) basis set. This correlation‐consistent nature of this basis set (cc‐pVTZ) includes higher angular‐momentum and diffuse functions (aug) to improve the description of electron correlation and noncovalent interactions such as hydrogen bonding, van der Waals forces, and π–π stacking. For ligand optimization, the B3LYP functional with the 6‐31G(d,p) basis set was used, including Grimme’s D3 dispersion correction and the Polarizable Continuum Model (PCM, water) to account for van der Waals and solvation effects. The self‐consistent‐field (SCF) convergence threshold was set to 10^−8^ a.u. to ensure accurate energy minimization [28].

Polarisation functions (denoted by *p*) increase the flexibility of the basis set by allowing orbitals to distort, thereby improving the accuracy of charge‐redistribution modelling and induced‐fit effects in ligand–receptor complexes. This feature is especially important when simulating interactions with charged amino‐acid residues within a binding site [29, 30]. Both solvation energy and ligand polarisation must be considered to obtain reliable estimates of binding energies in protein–ligand complexes, as demonstrated in previous studies on molecular recognition [31].

It is important to note that the computational model assumed fixed geometries and did not capture conformational flexibility. Solvent effects were treated with a distance‐dependent dielectric model that excludes explicit solvent molecules and ions.

Vibrational‐frequency analyses were performed to confirm that the optimised structures represented true local minima on the potential‐energy surface. The total energy of each molecule and its dipole moment were calculated to characterise electronic properties, with the dipole moment reflecting molecular polarity arising from charge separation.

DFT is widely used for molecular modelling and provides insights into electron distribution and related quantum descriptors. The calculations included the energies of the highest occupied molecular orbital (HOMO) and the lowest unoccupied molecular orbital (LUMO), the HOMO–LUMO gap (GAP), ionisation potential (I), electron affinity (*A*), chemical hardness (*η*), softness (*σ*), chemical potential (*μ*), electronegativity (*χ*) and electrophilicity index (*ω*). These descriptors were calculated using the following relationships [32]:

GAP=εHOMO−εLUMO;

I≈−εHOMO;

A≈−εLUMO;

$$\eta \approx ½\left(\text{εLUMO}-\text{εHOMO}\right)\approx ½\left(I-A\right);$$

σ=1/η;

$$\mu \approx ½\left(\text{εHOMO}+\text{εLUMO}\right)\approx -½\left(I+A\right);$$

$$\chi \approx -\mu \approx \left(I+A\right)/2;$$

$$\omega \approx \chi^{2}/\text{2η}$$

### 2.2 Lipinski Rule, Pharmacokinetics and Drug Likeness

Predicting drug‐likeness is crucial for identifying novel candidates and accelerating drug development. A structural and physicochemical analysis was conducted to illustrate each compound's similarity to established drugs, thereby supporting its potential as an alternative therapy [33]. ADMETlab 2.0 (https://admetmesh.scbdd.com/), pkCSM (https://biosig.lab.uq.edu.au/pkcsm/), vNN‐ADMET (https://vnnadmet.bhsai.org/vnnadmet/home.xhtml), admetSAR 3.0 (https://lmmd.ecust.edu.cn/admetsar3/), Deep‐PK (https://biosig.lab.uq.edu.au/deeppk/prediction), and PRED‐hERG (http://predherg.labmol.com.br/) were used to predict the ADMET profiles of the reported molecules. These platforms are widely employed in drug discovery to estimate pharmacokinetic and toxicity endpoints, and the predictions were interpreted according to standard threshold criteria.

**FIGURE 1:**
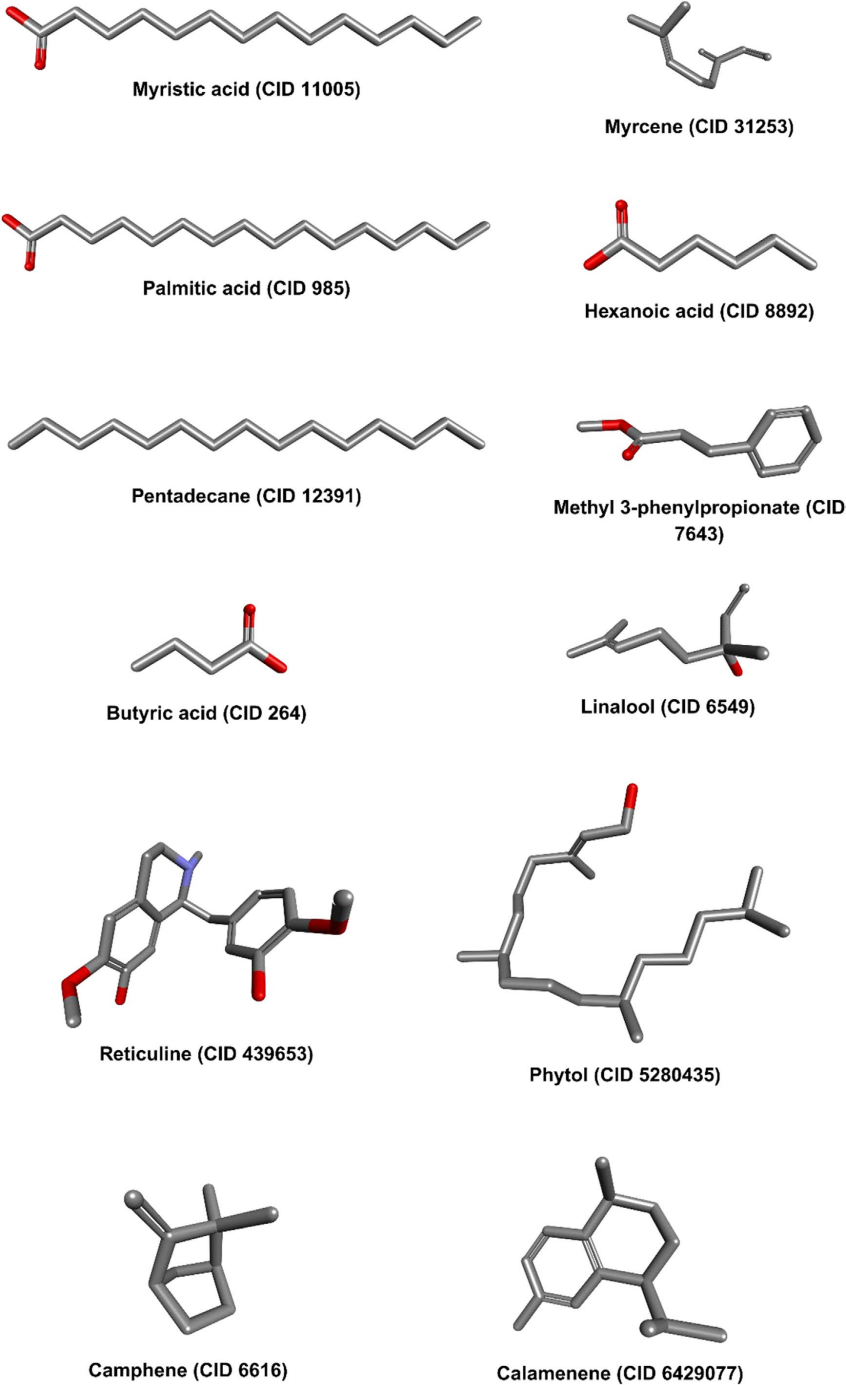
Schematic representation of the bioactive compounds from *Annona muricata* .

Lipinski's Rule of Five, formulated by Christopher Lipinski in 2004, remains a commonly applied guideline for assessing drug‐like properties of small molecules [34]. The SwissADME web tool (http://www.swissadme.ch/index.php) was used to evaluate compliance with Lipinski's criteria and other drug‐likeness metrics [35]. The molecular structures of the ligands are presented in Figure 1.

### 2.3 Molecular Docking

**FIGURE 2:**
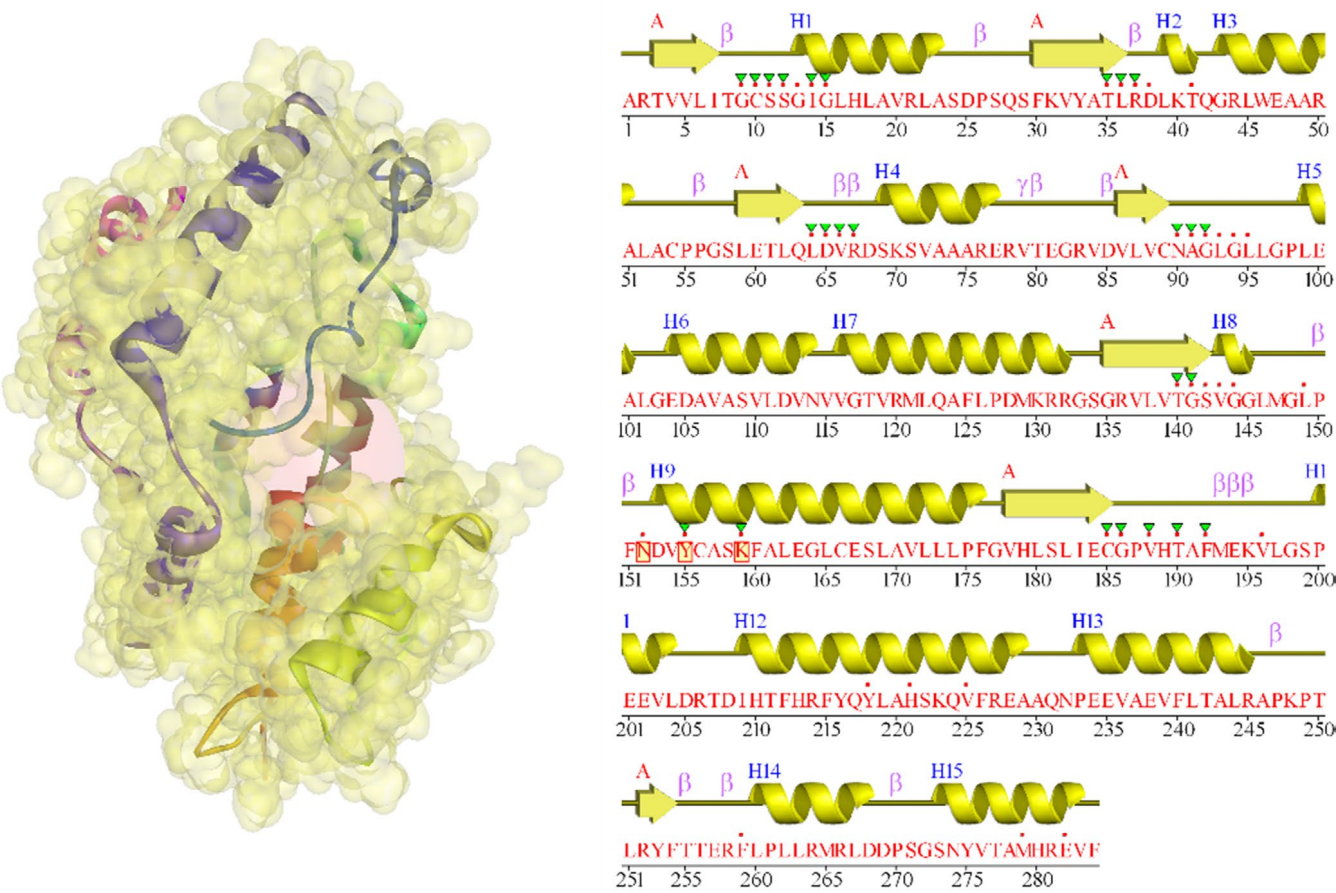
Schematic representation of the 17β‐HSD1, with the binding site highlighted as a red sphere. The van der Waals interaction regions are shown in transparent yellow (left), together with the motif topology of the same protein (PDB ID: 3HB5).

Following the completion of the structural, electronic, quantum‐descriptor and ADMET analyses of myristic acid, myrcene, palmitic acid, hexanoic acid, pentadecane, methyl 3‐phenylpropionate, butyric acid, linalool, reticuline, phytol, camphene and calamenene, we investigated their interactions with 17β‐hydroxysteroid dehydrogenase type 1 (17β‐HSD1), which was retrieved from the Protein Data Bank (PDB ID: 3HB5) (Figure 2).

To prepare the receptor for docking and correct crystallographic artefacts, we validated the structure with MolProbity (http://molprobity.biochem.duke.edu/) and generated docking files with AutoDockTools (ADT) (https://ccsb.scripps.edu/mgltools/downloads/) and Discovery Studio (https://www.3ds.com/products/biovia/discovery‐studio). The refinement protocol comprised: (i) removal of crystallographic water molecules to exclude non‐essential interactions; (ii) Ramachandran‐plot analysis, which showed that 92.2% of residues lay in favoured regions, confirming backbone integrity; (iii) correction of rotamer outliers—particularly for histidine, glutamine and asparagine side chains—to ensure proper hydrogen bonding; (iv) resolution of steric clashes to eliminate atomic overlaps; (v) adjustment of protonation states at physiological pH 7.4 to reflect correct tautomeric forms and interaction networks and (vi) energy minimisation with the CHARMm force field to relieve residual strain and improve overall geometry.

**FIGURE 3:**
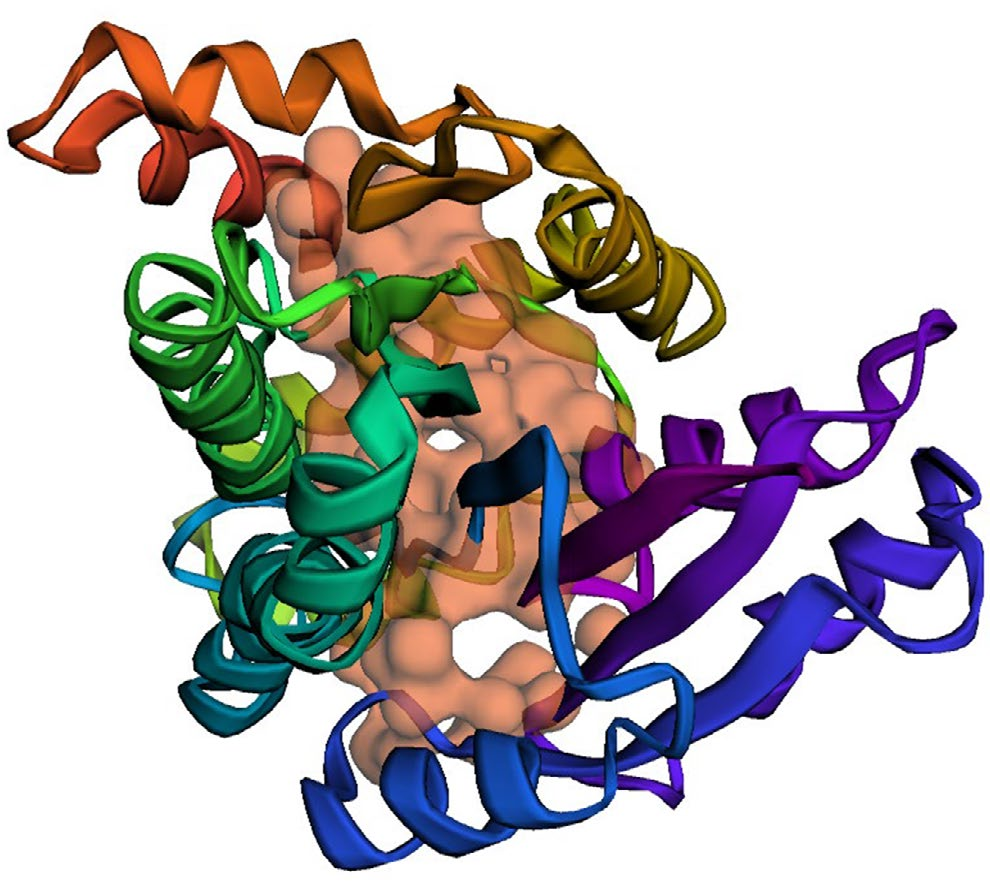
The ideal binding site for the enzyme 17β‐HSD1 is shown as an orange cloud within the enzyme (FPocketWeb 1.0.1 server).

The optimal binding site (Figure 3) was identified with FPocketWeb 1.0.1 (https://durrantlab.pitt.edu/fpocketweb/). Among the cavities detected, the one with the highest pocket score—indicating the greatest ligand‐binding potential—was selected and corresponded to the catalytic pocket of 17β‐HSD1.The docking grid was centered on the centroid of the native ligand at X = 10.5, Y = 24.3, Z = 18.7 Å, and a 40 × 40 × 40 Å³ box was defined to fully encompass the substrate‐binding region and adjacent residues. Docking was performed with AutoDock Vina 1.1.2 (https://vina.scripps.edu/), generating 50 poses per ligand with an exhaustiveness level of 25, a setting previously shown to balance accuracy and computational cost [36]. Epirubicin was used as the reference compound because it is an FDA‐approved anthracycline widely used in the treatment of estrogen‐dependent breast cancer, thus providing a clinically relevant benchmark for comparison with the phytochemicals. Validation of the docking protocol was performed by re‐docking the co‐crystallized ligand from PDB 3HB5, and superimposition of the docked and experimental poses yielded an RMSD = 1.2 Å, confirming the accuracy of the docking procedure.

To refine and validate the docking results, flexible docking was performed using HADDOCK 2.4 (https://rascar.science.uu.nl/haddock2.4/), which integrates ambiguous interaction restraints (AIRs), solvent refinement, and multistage molecular dynamics to improve accuracy [37]. HADDOCK comprises five steps. First, molecular topologies and structural parameters are generated, during which non‐polar hydrogens are removed to optimise performance. Second, rigid‐body docking (it0) samples random orientations and performs energy minimization to explore global binding modes. In this step, 1000 initial rigid‐body docking solutions were generated. Third, semi‐flexible simulated annealing (it1) allows residues within 5.0 Å of the ligand to adjust conformations through stepwise annealing in vacuo while preserving the overall protein fold. The best 200 docking solutions from the previous stage were refined in this step, with the ligand kept fully flexible. Fourth, final refinement is carried out in explicit solvent: surface‐contact and center‐of‐mass restraints stabilise the complex during short molecular‐dynamics (MD) simulations. A total of 1250 MD steps were run at 300 K, with positional restraints on heavy atoms not involved in key interactions, followed by stepwise cooling to 200 K and 100 K to optimise side‐chain conformations at the interface. HADDOCK scores were calculated using its default scoring function, which includes van der Waals, electrostatic, desolvation, and restraint energy terms. All other parameters were kept at their default values unless otherwise specified.

Finally, the resulting structures were clustered by root mean square deviation (RMSD) using a 1.5 Å cutoff and ranked by the HADDOCK score, which combines van der Waals, electrostatic, desolvation, and restraint energies. The most favourable complex for each ligand underwent additional validation with the PRODIGY server, which predicted Gibbs free energy (ΔG) and atom–atom contact values to support the strength and specificity of the interactions [38, 39].

### 2.4 Molecular Dynamics (MD) Simulation

The validity of the docking results was confirmed by MD simulations, an essential component of in silico workflows for verifying protein–ligand stability and analysing the fluctuations and structural adaptations of a complex as it relaxes toward a stable configuration [40]. MD simulations (100 ns each) were carried out for the top two ligands, reticuline and calamenene, together with epirubicin as the standard compound. All simulations employed the CHARMM36 force field within the GROMACS 2020 package. Ligand and protein topologies were generated through the CHARMM‐GUI server, which assigned CHARMM36‐compatible parameters using the CGenFF (version 4.6) program [41, 42, 43].

Each ligand–protein complex was placed in a rectangular box with a 10 Å buffer in every direction and solvated with TIP3P water. System neutrality was achieved by adding Na^+^ and Cl^−^ ions, and the structure was energy‐minimised using the steepest‐descent algorithm. Equilibration was performed at 310 K for 10 ps (5000 steps), first under a constant‐volume, constant‐temperature (NVT) ensemble, followed by a constant‐pressure, constant‐temperature (NPT) ensemble [44, 45].

The LINCS algorithm constrained bonds involving hydrogens, allowing a 2‐fs integration time step [46]. Van der Waals interactions were treated with a switching function between 12 and 14 Å, using a 14 Å cutoff. Long‐range electrostatic interactions were calculated with the particle‐mesh Ewald (PME) method, employing a maximum grid spacing of 1.2 Å [47]. PME calculations were executed at every step, the temperature was maintained at 310 K, and the barostat preserved the pressure at 1 bar.

Binding free energies were estimated using the MM‐PBSA approach with the g_mmpbsa script interfaced with GROMACS 2021. For the polar solvation energy, the Poisson–Boltzmann model was used with a solute dielectric constant of 1 and a solvent dielectric of 80 (water). A total of 100 snapshots were extracted from the last 20 ns of the MD trajectory at 200 ps intervals for analysis. After completion of the production runs, trajectories were recentered and analysed with built‐in GROMACS utilities and VMD to extract RMSD, RMSD, radius of gyration (Rg), hydrogen‐bond counts, principal component analysis (PCA) and dynamic cross‐correlation matrix (DCCM) [48, 49].

## 3 Results and Discussion

### 3.1 Quantum Analysis

Reticuline exhibited the most exergonic total energy (−1093.99 Ha), whereas butyric acid displayed the least exergonic value (−307.86 Ha). In terms of dipole moment, reticuline again ranked highest (4.82 D), while pentadecane showed the lowest polarity (0.10 D) (Table 1).

**TABLE 1:** Quantum energies of the compounds. Total energy is in Hartree (Ha) and Dipole in Debye (*D*).

Quantum energies			
No	Ligands	Total energy (Ha)	Dipole (*D*)
01	Myristic acid	−701.19	2.05
02	Myrcene	−390.84	0.25
03	Palmitic acid	−779.85	2.05
04	Hexanoic acid	−386.53	2.05
05	Pentadecane	−591.19	0.10
06	Methyl 3‐phenylpropionate	−539.00	2.27
07	Butyric acid	−307.86	2.05
08	Linalool	−467.32	2.25
09	Reticuline	−1093.99	4.82
10	Phytol	−861.88	2.40
11	Camphene	−390.86	0.98
12	Calamenene	−585.15	0.52

The frontier‐orbital energies of the ligands range from a HOMO energy of −7.92 eV for butyric acid to −5.69 eV for the benzyl‐isoquinoline alkaloid reticuline, indicating that the latter can readily donate electrons—a property often associated with the antioxidant activity of such molecules [50]. In parallel, the LUMO energies span from −1.06 eV for myrcene to 0.98 eV for pentadecane. Evaluating these frontier orbitals offers significant insight into the molecular interactions and chemical reactivity of the compounds [51, 52].

Consequently, the HOMO–LUMO gap narrows to 5.06 eV for reticuline but widens to 8.84 eV for pentadecane. A large band gap generally suggests high kinetic stability and low chemical reactivity [53].

Ionisation potentials (I) follow the HOMO trend and range from 5.69 eV (reticuline) to 7.92 eV (butyric acid), underscoring the relative ease of oxidising the alkaloid—consistent with its high HOMO energy and small band gap [54].

Electron affinities (A) peak at 1.06 eV for myrcene and drop to −0.98 eV for pentadecane, emphasising myrcene's ability to attract electrons in electrophilic reactions while highlighting the weak electron‐attracting nature of linear alkanes.

These I and A values yield global‐hardness minima (*η*) of 2.53 eV for reticuline and maxima of 4.42 eV for pentadecane. Within the Parr–Pearson HSAB framework, lower hardness corresponds to greater chemical adaptability and polarizability [55], characteristics that strongly influence chemical–biological interactions [56].

Softness (*σ* = 1/*η*) is 0.40 eV^−1^ for reticuline but diminishes to 0.23 eV^−1^ for pentadecane, indicating the pronounced electronic flexibility of the alkaloid compared with the rigid hydrocarbon chain.

Chemical potentials (*μ*) range from −3.16 eV (reticuline) to −4.04 eV (butyric acid), whereas electronegativity (*χ*) displays the reverse order (3.16 eV for reticuline and 4.04 eV for myristic acid). Ligands with high chemical potential, such as reticuline, are more prone to electron loss, whereas those with high electronegativity—that is myristic acid—have a greater tendency to attract electron density [57].

Finally, electrophilicity indices (*ω*) vary from 12.61 eV for reticuline to 31.66 eV for butyric acid. Electrophilicity has been correlated with the environmental biodegradability of drugs [58], suggesting that butyric acid may exhibit lower bioaccumulation. All calculated values are summarised in Table 2.

**TABLE 2:** Quantum chemical descriptors of the ligands, including ionisation potential (I), electron affinity (A), chemical hardness (ɳ), softness (*σ*), chemical potential (*μ*), electronegativity (*χ*) and electrophilicity index (*ω*). All values are given in electron‐volts (eV).

No	Quantum chemical descriptors* **										
	Compounds	HOMO	LUMO	GAP	*I*	*A*	*η*	*σ*	*μ*	*χ*	*ω*
01	Myristic acid	−7.89	−0.16	7.73	7.89	0.16	3.86	0.26	−4.03	4.03	31.34
02	Myrcene	−6.42	−1.06	5.36	6.42	1.06	2.68	0.37	−3.74	3.74	18.76
03	Palmitic acid	−7.86	−0.16	7.70	7.86	0.16	3.85	0.26	−4.01	4.01	31.01
04	Hexanoic acid	−7.89	−0.16	7.73	7.89	0.16	3.86	0.26	−4.03	4.03	31.34
05	Pentadecane	−7.86	0.98	8.84	7.86	−0.98	4.42	0.23	−3.44	3.44	26.20
06	Methyl 3‐phenylpropionate	−6.88	−0.54	6.34	6.88	0.54	3.17	0.32	−3.71	3.71	21.87
07	Butyric acid	−7.92	−0.16	7.76	7.92	0.16	3.88	0.26	−4.04	4.04	31.66
08	Linalool	−6.45	−0.16	6.29	6.45	0.16	3.14	0.32	−3.31	3.31	17.18
09	Reticuline	−5.69	−0.63	5.06	5.69	0.63	2.53	0.40	−3.16	3.16	12.61
10	Phytol	−6.64	−0.03	6.61	6.64	0.03	3.31	0.30	−3.33	3.33	18.37
11	Camphene	−6.69	0.19	6.88	6.69	−0.19	3.44	0.29	−3.25	3.25	18.20
12	Calamenene	−6.37	−0.46	5.90	6.37	0.46	2.95	0.34	−3.42	3.42	17.22

### 3.2 Lipinski Rule, Pharmacokinetics and Drug Likeness

#### 3.2.1 Lipinski Rule and Drug Likeness

Drug‐likeness plays a crucial role in the development of potential medicines, encompassing both the optimization of a chemical entity and the evaluation of its pharmacokinetic behaviour. It reflects the interplay of molecular and structural features such as molecular weight (MW), lipophilicity (log Po/w), the number of hydrogen‐bond acceptors and donors and predicted bioavailability. This overall balance is commonly assessed with computational filters embodied in Lipinski's rule of five [34].

**FIGURE 4:**
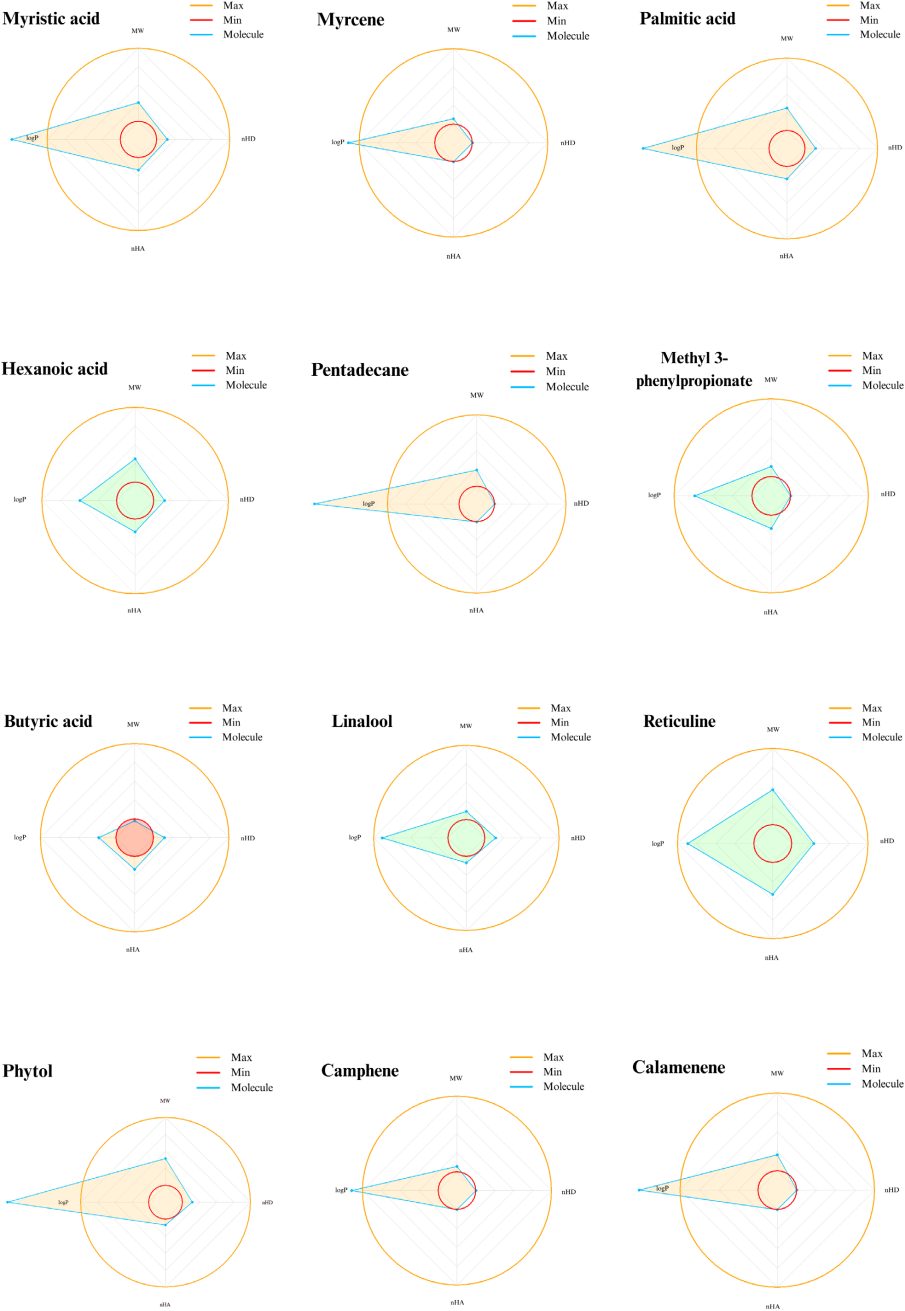
Graphical representation of the results obtained for the physicochemical properties of the ligands.

In the present study, all compounds displayed MW values below 500 g mol^−1^ and, with few exceptions, complied with Lipinski's criteria. Palmitic acid, pentadecane and phytol exceeded the log P threshold of 5 and therefore violated one Lipinski descriptor, whereas the remaining phytochemicals fully satisfied the rule. Detailed values for each *Annona muricata* metabolite are listed in Table 3, and Figure 4 illustrates the drug‐likeness profile of every ligand.

**TABLE 3:** Properties of the ligands according to Lipinski's rule, molecular weight (MW) in g/mol, hydrogen bond acceptor (HBA), hydrogen bond donor (HBD) and partition coefficient LogP.

Name	MW	HBA	HBD	LogP	Lipinski rule	
					Result	Violation
Myristic acid	228.37	2	1	4.45	Yes	0
Myrcene	136.24	0	0	3.43	Yes	0
Palmitic acid	256.42	2	1	5.20	Yes	1
Hexanoic acid	116.16	2	1	1.47	Yes	1
Pentadecane	212.41	0	0	6.06	Yes	1
Methyl 3‐phenylpropionate	164.20	2	0	2.21	Yes	0
Butyric acid	88.11	2	1	0.68	Yes	0
Linalool	154.25	1	1	2.66	Yes	0
Reticuline	329.39	5	2	2.60	Yes	0
Phytol	296.53	1	1	6.22	Yes	1
Camphene	136.23	0	0	3.43	Yes	1
Calamenene	202.34	0	0	4.57	Yes	1

#### 3.2.2 ADME

The Caco‐2 permeability assays suggested generally favourable absorption profiles, with values ranging from 0.919 (~8.3 × 10^−6^ cm·s^−1^) to 1.566 (~36.8 × 10^−6^ cm·s^−1^). Hexanoic acid showed the highest permeability (1.566), indicating a superior capacity to cross the intestinal barrier and, consequently, more efficient absorption into the systemic circulation, which can enhance therapeutic effectiveness [59]. By contrast, reticuline presented the lowest permeability (0.919), suggesting that its oral bioavailability may be limited; this finding could necessitate structural modification or specialised formulation to improve absorption [60].

Predictions of P‐glycoprotein (Pgp) interactions revealed distinct behaviours among the compounds. Reticuline was identified as a Pgp substrate but not an inhibitor, indicating its active transport out of intestinal epithelial cells; such efflux can reduce systemic exposure, compromise therapeutic outcomes, and contribute to drug resistance [61]. Conversely, calamenene was predicted to be a Pgp inhibitor without acting as a substrate, which could help increase the bioavailability of co‐administered Pgp‐substrate drugs by limiting their efflux [62]. Indeed, calamenene has been predicted to inhibit this transporter without itself being transported, potentially enhancing the bioavailability of other substrates when co‐administered [62].

Distribution properties—especially plasma‐protein binding (PPB) and blood–brain‐barrier (BBB) permeability—directly influence the pharmacodynamics of active ingredients [63]. Palmitic acid and phytol showed the highest PPB, indicating a lower free‐drug fraction that could limit tissue penetration yet possibly prolong plasma half‐life; these findings align with published studies emphasising the high protein affinity and low bioavailability of such molecules [64, 65]. Conversely, butyric acid had the lowest PPB, indicating a higher free fraction available for tissue interaction and pharmacological activity. BBB‐permeability assessments varied, with pentadecane exhibiting the highest permeability. High BBB permeability could pose neurological risks, given reports of adverse effects associated with drugs that, although not intended for the nervous system, cross the BBB and lead to neurological disorders and encephalopathies [66, 67]. In contrast, the minimal BBB permeability predicted for butyric acid is advantageous because it reduces the likelihood of central‐nervous‐system adverse effects and makes systemic administration to peripheral tissues, such as breast cancer tumours, safer.

Cytochrome P450 (CYP450) enzymes significantly influence drug metabolism [68]. In our predictions, reticuline and calamenene were classified as potent CYP3A4 substrates, which may reduce their bioavailability and therapeutic efficacy owing to rapid metabolism and may require dosage adjustment to maintain therapeutic concentrations [69]. Methyl 3‐phenylpropionate was predicted to inhibit both CYP1A2 and CYP2C19, a property that could lead to bioaccumulation of co‐administered drugs such as clozapine [70].

Pentadecane exhibited the shortest predicted half‐life, which may result in rapid elimination from the systemic circulation and potentially reduced therapeutic duration and efficacy unless compensated by dose adjustments. In contrast, reticuline had a long‐predicted half‐life, suggesting prolonged systemic retention and potentially better therapeutic outcomes through sustained bioactivity in target tissues, although this may also increase the risk of toxicity [71].

#### 3.2.3 Toxicity

The prediction models PRED‐hERG and pkCSM indicated that the investigated compounds present distinct toxicological profiles, highlighting areas of concern for pre‐clinical safety assessment. With regard to cardiotoxicity, toxicity that affects the heart and can result in arrhythmia, myocardial hypertrophy or infarction [72], pentadecane was predicted to block the hERG channel in some binary models, whereas methyl 3‐phenylpropionate and calamenene were consistently classified as moderate blockers in multiclass predictions. In addition, phytol and reticuline were identified as potential hERG II inhibitors, implying a risk of QT prolongation that may induce polymorphic ventricular tachycardia [73].

Drug‐induced liver injury (DILI) remains a leading cause of compound discontinuation during clinical development and after approval [74]. Liver‐related toxicity was predicted for myrcene, which was associated with DILI in at least one model, and for methyl 3‐phenylpropionate, which yielded positive results in three independent tools (vNN‐ADMET, admetSAR 3.0 and pkCSM). Because DILI can involve metabolic, immunological and genetic pathways [75], its impact may be exacerbated in patients with pre‐existing liver disorders or severe concomitant dermatological reactions [76]. Reticuline triggered a neurotoxicity alert in admetSAR 3.0, possibly owing to mitochondrial dysfunction that could lead to physiological disturbances and brain damage [77]. Phytol was the only compound flagged by a genotoxic‐carcinogenicity rule, and myrcene showed carcinogenic potential in two models (Deep‐PK and ADMETlab 2.0). As carcinogens may damage the genome and disrupt cellular metabolism, such findings warrant experimental verification, especially because the compounds are candidates for cancer therapy [78].

**FIGURE 5:**
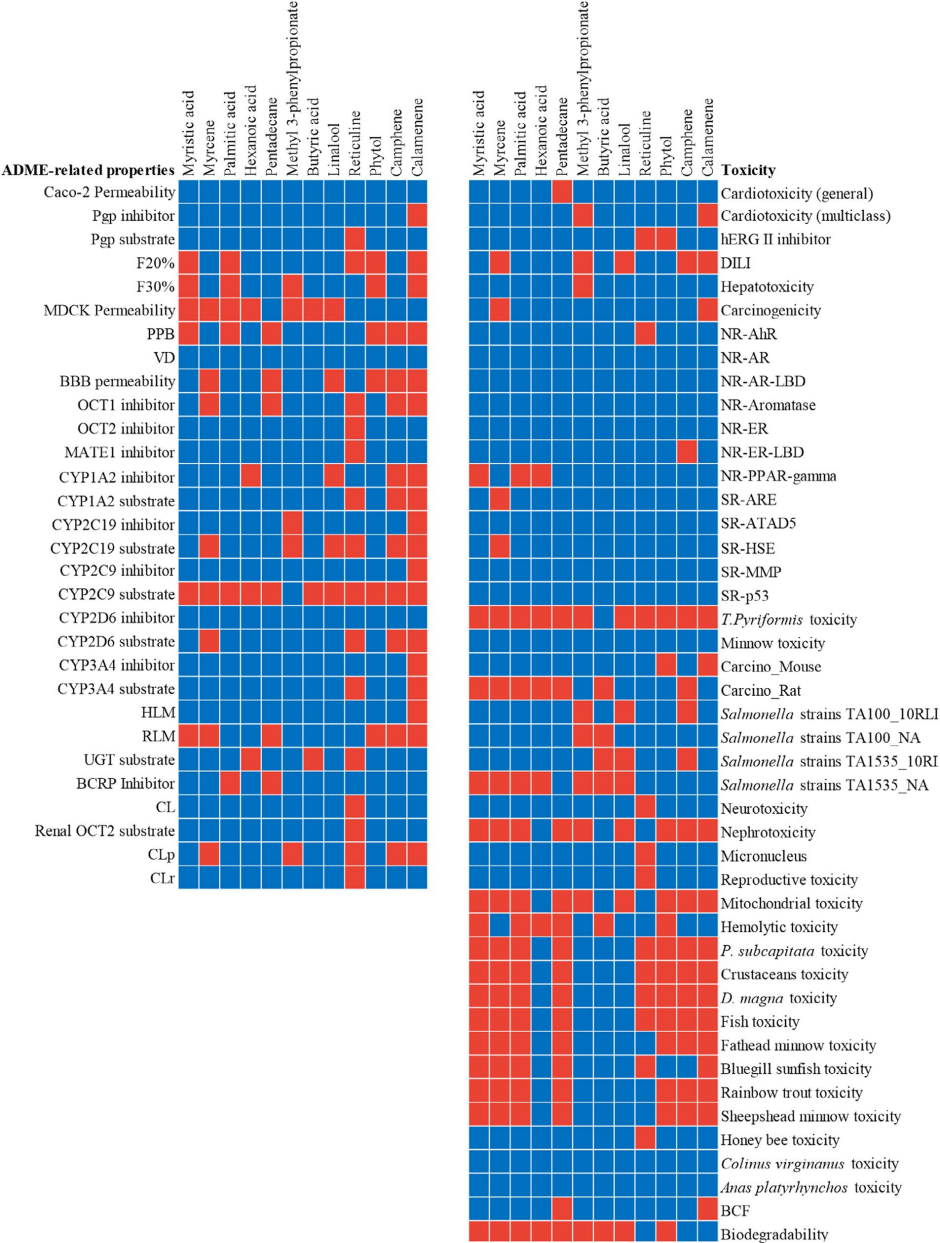
Comparative heatmap of the ADMET profiles for the analysed compounds. The left panel represents ADME‐related properties, while the right panel shows the predicted toxicity endpoints. Blue cells indicate favourable properties, while red cells represent unfavourable properties, including poor ADME behaviour or predicted toxicological risks. For CYP‐related properties, blue indicates activity (either as a substrate or inhibitor), and red indicates inactivity.

Environmental‐toxicity assessments further underscored the complexity of the safety profiles. Predictive biodegradation models suggested that most compounds are poorly degradable, raising the possibility of bioaccumulation [79]. Moreover, Deep‐PK and admetSAR 3.0 projected that myristic acid, palmitic acid, pentadecane and phytol could be toxic to aquatic organisms such as fish, *P. subcapitata* and crustaceans, indicating a risk of ecological imbalance, e.g., feminization of male fish, avian mortality by poisoning and disruption of food chains [80]. Figure 5 provides an overview of all ADMET properties.

### 3.3 Molecular Docking and Interaction Analysis

Molecular docking was conducted to evaluate the interaction profiles and binding affinities of reticuline and calamenene with the 17β‐HSD1 enzyme, as they exhibited higher binding affinities (kcal mol^−1^) than the other ligands in the initial screening (Table 4).

**TABLE 4:** Binding affinity against targeted receptor.

N°	Phytochemicals present in *Annona muricata*	Binding affinity (kcal/mol)
1.	Myristic acid	−5.4
2.	Myrcene	−5.0
3.	Palmitic acid	−4.5
4.	Hexanoic acid	−4.6
5.	Pentadecane	−5.1
6.	Methyl 3‐phenylpropionate	−5.9
7.	Butyric acid	−4.1
8.	Linalool	−4.7
9.	Reticuline	−8.4
10.	Phytol	−5.3
11.	Camphene	−5.1
12.	Calamenene	−7.7
13.	Standard epirubicin hydrochloride	−5.7

Reticuline achieved a highly favourable HADDOCK score of −46.02 kcal mol^−1^, driven chiefly by strong van der Waals interactions (Evdw = −33.02 kcal mol^−1^) and substantial electrostatic contributions (Eelec = −30.40 kcal mol^−1^). The ligand displayed moderate desolvation energy (−6.92 kcal mol^−1^), reflecting a balanced contribution of hydrophobic and polar contacts. Its predicted binding affinity (ΔGprediction = −8.23 kcal mol^−1^) is consistent with an electron‐rich aromatic scaffold that facilitates interactions with residues in the active site.

By contrast, calamenene yielded a lower HADDOCK score of −34.67 kcal mol^−1^, characterised mainly by hydrophobic interactions, as indicated by a more negative desolvation energy (−16.66 kcal mol^−1^) and moderate van der Waals contributions (−17.90 kcal mol^−1^). The negligible electrostatic term (−0.56 kcal mol^−1^) underscores its reliance on hydrophobic complementarity rather than polar contacts, a behaviour previously reported for similar scaffolds [81]. The predicted binding affinity (ΔGprediction = −7.91 kcal mol^−1^) supports a moderately efficient interaction compared with reticuline.

Because of its small HOMO–LUMO gap, reticuline exhibits greater electronic reactivity and conformability, promoting charge transfer and hydrogen bonding—features that align with the strong electrostatic interactions observed in docking. Its high electrophilicity index and lower global hardness further enhance its capacity for polar interactions and conformational fitting within the binding cavity, thereby increasing complex stability. Calamenene, characterised by higher lipophilicity and limited electronic reactivity (fewer polar contacts and minimal electrostatic contribution), interacts predominantly through hydrophobic pathways. Its higher internal binding energy (173.54 kcal mol^−1^) compared with reticuline (115.15 kcal mol^−1^) reflects the energetic cost of structural reorganisation upon binding, potentially limiting pharmacological efficacy.

**FIGURE 6:**
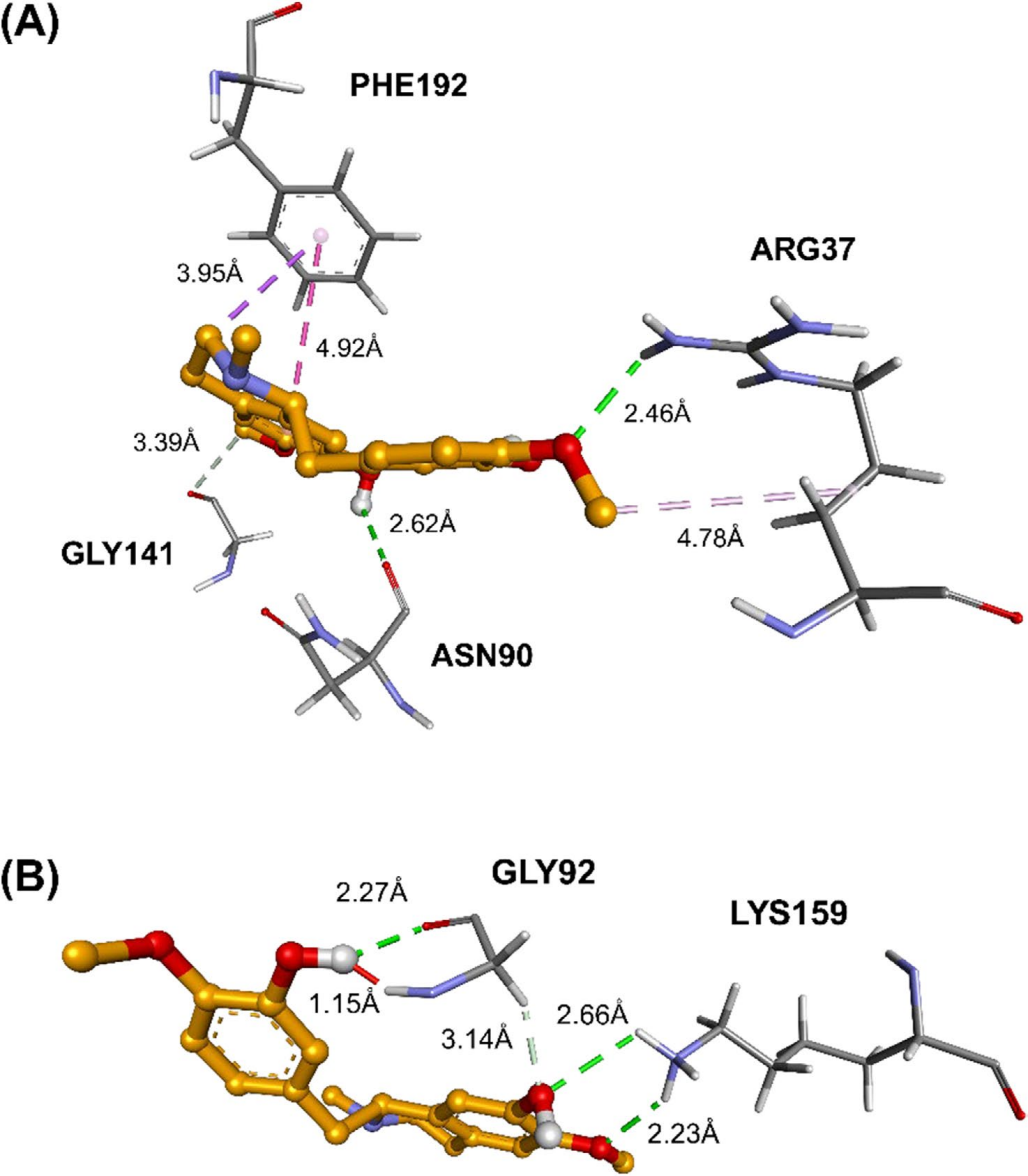
Molecular interactions of the reticuline (orange) with amino acids from the enzyme 17β‐HSD1. Hydrophobic π‐alkyl interactions are shown in light purple, T‐shaped π–π interactions in medium purple and π–*σ* interactions in dark purple. Conventional hydrogen bonds are shown in green, while carbon‐hydrogen bonds are shown in white. Unfavourable interactions are indicated in red. (A) and (B) represent the same ligand from different perspectives.

Docking analysis revealed that the binding affinity of reticuline for 17β‐HSD1 is largely governed by an extensive network of polar and π‐directed contacts in the active site (Figure 6). Hydrogen bonds were formed with GLY92 (two contacts), GLY141, LYS159 (two contacts), ASN90 and ARG37, anchoring the ligand in a catalytically competent orientation. In addition, PHE192 engaged the aromatic scaffold of reticuline through π–π stacking and π–sigma interactions, while ARG37 contributed an additional hydrophobic alkyl contact. Collectively, these interactions enhance complex stability and may account for the significant inhibitory potential of this alkaloid.

**FIGURE 7:**
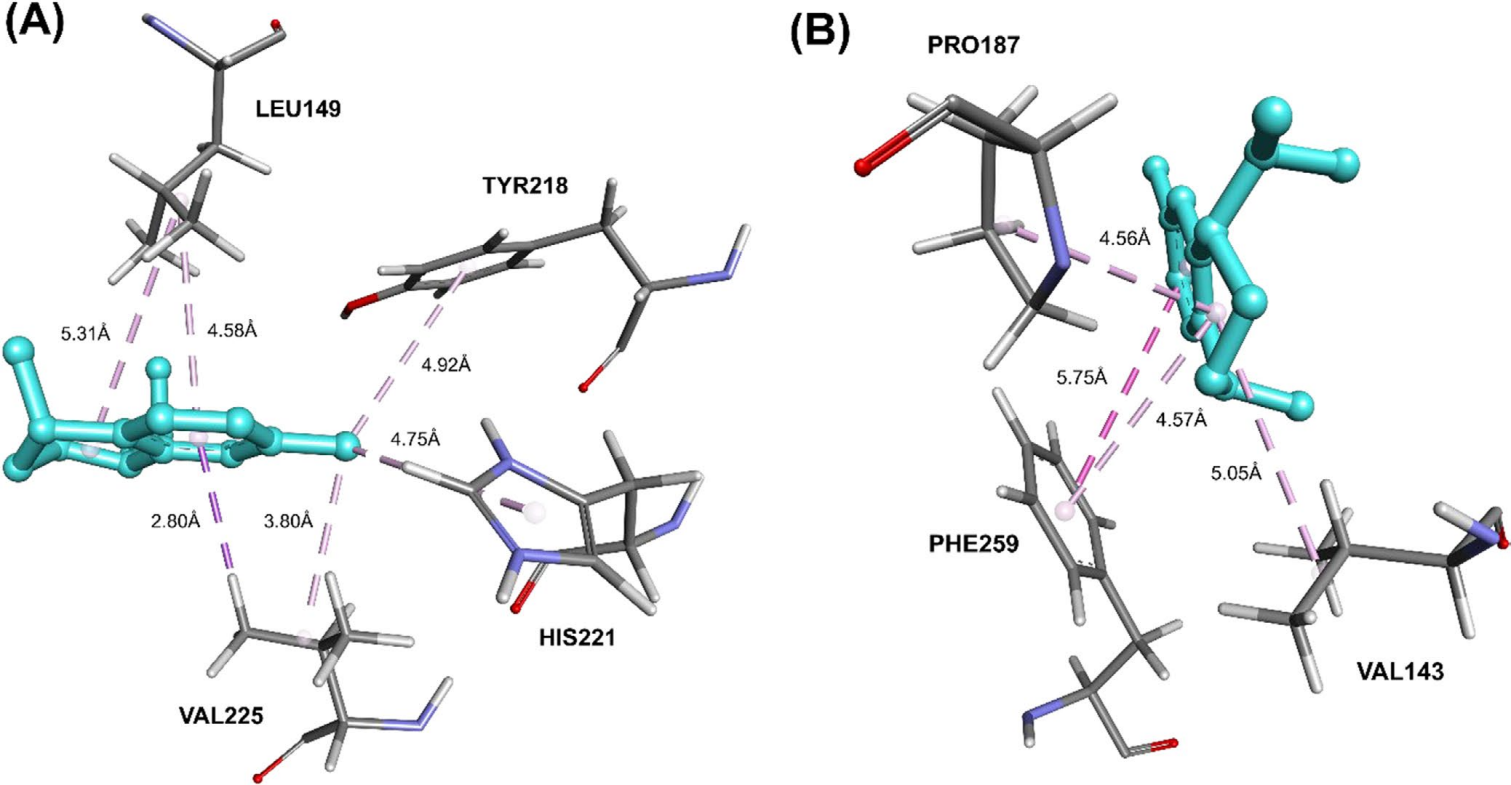
Molecular interactions of the calamenene (cyan) with amino acids from the enzyme 17β‐HSD1. Hydrophobic π‐alkyl interactions are shown in light purple, T‐shaped π–π interactions in medium purple and π–*σ* interactions in dark purple. (A) and (B) represent the same ligand from different perspectives.

In contrast, calamenene displayed a predominantly hydrophobic binding mode, with no hydrogen bonds detected (Figure 7). Its aromatic core established π–π stacking with PHE259 and a π–sigma contact with VAL225, while alkyl interactions with VAL143, PRO187, PHE259, LEU149 (two contacts), VAL225, HIS221 and TYR218 created an apolar microenvironment that favours ligand accommodation. This ensemble of van der Waals and π‐mediated contacts compensates for the absence of polar interactions, yielding a stable and energetically favourable complex between calamenene and 17β‐HSD1. Tables 5 and 6 summarise the docking‐refinement results.

**TABLE 5:** Energies obtained from HADDOCK and Prodigy Webserver for the complexes 17β‐HSD1 with reticuline and 17β‐HSD1 with calamenene.

Energies	17β‐HSD1 with reticuline	17β‐HSD1 with calamenene
HADDOCK score1	−46.02 (−46.02 to −34.91)	−34.67 (−34.67 to −26.98)
RMSD from the overall lowest‐energy structure	0 (0 to 0.762)	0 (0 to 0.779)
Desolvation energy (Edesolv)	−6.92 (−7.09 to −3.42)	−16.66 (−16.66 to −11.64)
Van der Waals energy (Evdw)	−33.02 (−33.3 to −25.99)	−17.9 (−19.25 to −14.91)
Electrostatic energy (Eelec)	−30.4 (−38.59 to −23.11)	−0.56 (−3.08 to 8.86)
Ambiguous interaction restraints energy (Eair) 2	No Eair was calculated for this run	No Eair was calculated for this run
ΔGprediction (Kcal/mol) 3	−8.23	−7.91
ΔGscore (Kcal/mol) 4	127.84	115.91
Internal energy free molecules	−10394.2	−10935.7
Internal energy complex	−10208.7	−10,727
Binding energy	115.15	173.54

**TABLE 6:** Number of atom–atom interactions nearby (within at 10.5 Å distance) between reticulin and calamenene with the enzyme 17β‐HSD1.

Interactions	17β‐HSD1 with reticuline	17β‐HSD1 with calamenene
CC	1993	1886
CO	1064	463
CN	667	399
CX	24	60
OO	136	0
OX	4	0
NO	159	0
NN	32	0
NX	2	0
XX	0	0

### 3.4 Molecular Dynamic Simulation Result Analysis

#### 3.4.1 Evaluating Stability Through RMSD Analysis

RMSD is an essential metric for assessing structural stability: high RMSD values signal substantial instability and indicate conformational changes in the molecule under study. A key parameter for analysing a protein–ligand complex is the RMSD of the protein‐backbone Cα atoms, which describes overall conformational stability during the simulation [82]. We plotted RMSD values for the backbone atoms extracted from the trajectories to evaluate stability. The 100‐ns simulation revealed continuous dynamics for all complexes.

**FIGURE 8:**
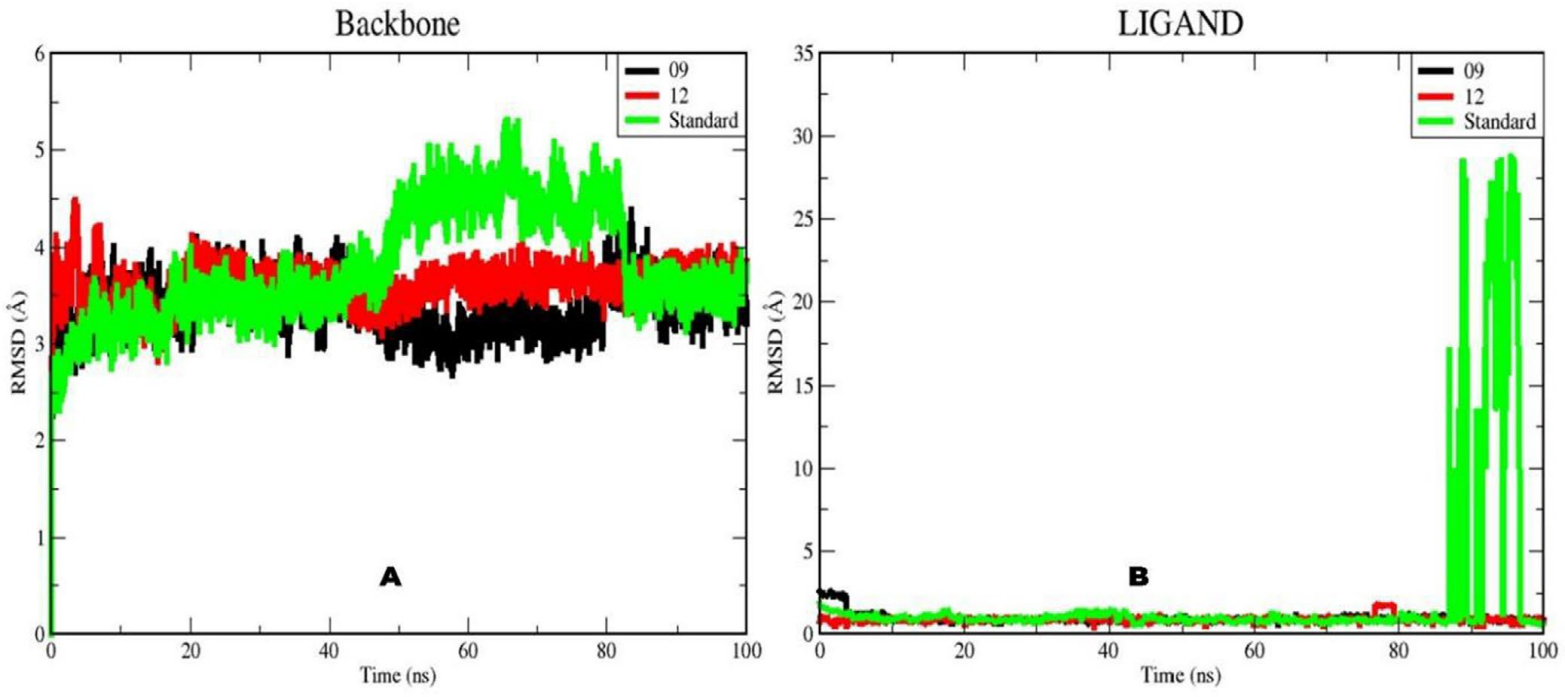
RMSD analysis of protein backbones and ligand complexes in MD simulation for production. (A) RMSD for backbone atoms of 09, 12 and standard protein. (B) RMSD for ligand systems of 09, 12 and standard protein.

Figure 8A shows the RMSD traces of the protein–ligand complex 09 (17β‐HSD1 with reticuline; black), complex 12 (17β‐HSD1 with calamenene; red) and the standard complex (17β‐HSD1 with epirubicin; green). All three complexes exhibited an increase in backbone RMSD within the first 5 ns, a rise that was particularly marked for complex 12. A brief drop followed, after which RMSD increased slightly until ≈40 ns. Between 40 and 85 ns, the RMSD of the standard system rose by 2.5 Å to its maximum [83, 84]. By comparison, complex 12 rose by only 0.5 Å and then increased steadily to the end of the run, whereas complex 09 showed minimal fluctuations over this interval. Although a pronounced rise occurred for complex 09 at 85 ns, all three complexes remained highly stable from that point until the simulation ended. Final RMSD values for backbone‐09, backbone‐12 and the standard were 3.4 Å, 3.6 Å and 3.8 Å, respectively; because all values remained below 5 Å, the complexes can be regarded as stable.

Figure 8B presents RMSD values for the ligands themselves (reticuline, calamenene and epirubicin) in complex with the protein. Average RMSD values were 0.684 Å for reticuline, 0.657 Å for calamenene and 2.215 Å for the standard. Ligand‐09 showed fluctuations in the first 5 ns of the trajectory, followed by a 1.5 Å drop in the RMSD value that remained steady for 10 ns. The RMSD value reached around 1 Å and remained steady throughout the 100 ns simulation period. This shows that Ligand‐09's contacts were unbroken during the simulation.

The Ligand‐12 molecule exhibited minimal changes from the start of the simulation until 75 ns, but observed a substantial rise in RMSD value between 75 and 77 ns. However, following this peak, the RMSD number dropped again and remained fairly steady until the end of the simulation. Furthermore, the trajectories were evaluated with the VMD software, and the results revealed that ligands 09 and 12 were not moving outside the protein domain, indicating that they remained within the binding region.

The RMSD examination of the ligands revealed that ligand‐09 and ligand‐12 did not change their binding orientation during the simulation. On the other hand, although the standard ligand compound exhibited similar behaviour to the other two compounds up to 85 ns into the simulation, the RMSD value showed a significant increase in the last 15 ns of the simulation. This increase confirms that the standard ligand exhibits multiple binding orientations and that this ligand changed its position throughout the MD simulations, not only leaving the binding site but also exhibiting the weakest interaction with the target protein.

#### 3.4.2 Root Mean Square Fluctuations

**FIGURE 9:**
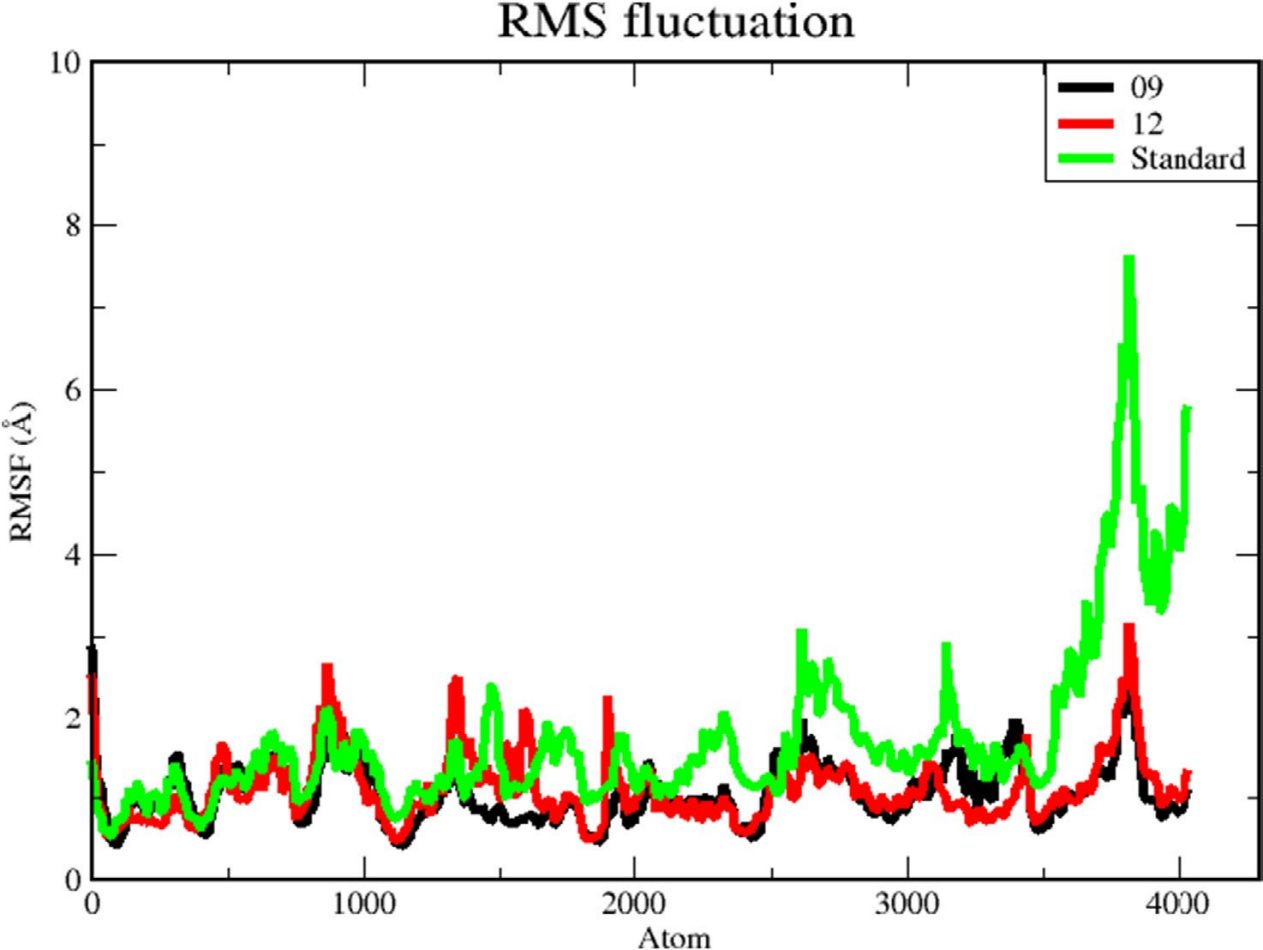
RMSF graph of a complex protein backbones. The RMSF of the protein‐reticuline backbone is depicted in black. The protein‐calamenene backbone RMSF is depicted in red. The standard backbone RMSF is depicted in green.

The root mean square fluctuation (RMSF) is used to measure the variability or flexibility of atoms in a molecular structure over time, particularly in molecular‐dynamics simulations. It provides insight into how much each atom in a molecule deviates from its average position during the simulation [85, 86]. Accordingly, RMSF data for the protein backbones were plotted to visualise the average fluctuation of all amino‐acid residues, as depicted in Figure 9 for the 100 ns MD trajectory.

Moreover, RMSF values serve as important indicators for evaluating the role of specific protein residues in preserving the structural integrity of a ligand–protein complex. A higher RMSF value implies a greater degree of flexibility, whereas a lower value denotes a more stable region. Therefore, a larger number of high‐RMSF residues reflect greater flexibility, which may enhance the likelihood of interactions with ligand molecules. Conversely, reduced RMSF is associated with diminished flexibility, consequently leading to a decreased potential for interactions.

The RMSF plot indicates that the standard compound shows the highest RMSF values and presents a notably greater number of significant peaks compared with the other compounds. All eight residues whose RMSF exceeds 4 Å in the standard complex are positioned within the protein's binding site.

Furthermore, the plot reveals significant peak changes at ARG264, MET265, ARG266, LEU267, ASP268, ASP269, PRO270 and PHE284. The higher peaks observed for the standard compound indicate that the interaction between the protein and ligand is less stable than in the other complexes.

The amino acids exhibiting marked fluctuations in both reticuline and calamenene complexes are located in identical regions. In addition, the calamenene complex displays higher RMSF values than reticuline, suggesting a less stable binding affinity. The RMSF plot of the protein–ligand complexes (Figure 9) also shows a notable rise at N‐terminal residues, which correspond to the terminal region of the protein. The ALA1 residue at the N‐terminus displays considerably larger fluctuations than the other residues, particularly in the reticuline and calamenene trajectories, most likely owing to its location near the flexible terminus.

Moreover, pronounced disparities are evident at the C‐terminus among the compounds. The higher C‐terminal RMSF values in the standard complex, relative to the other two, suggest that ligand binding weakens interactions in this complex, thereby increasing RMSF.

#### 3.4.3 Rg Analysis

**FIGURE 10:**
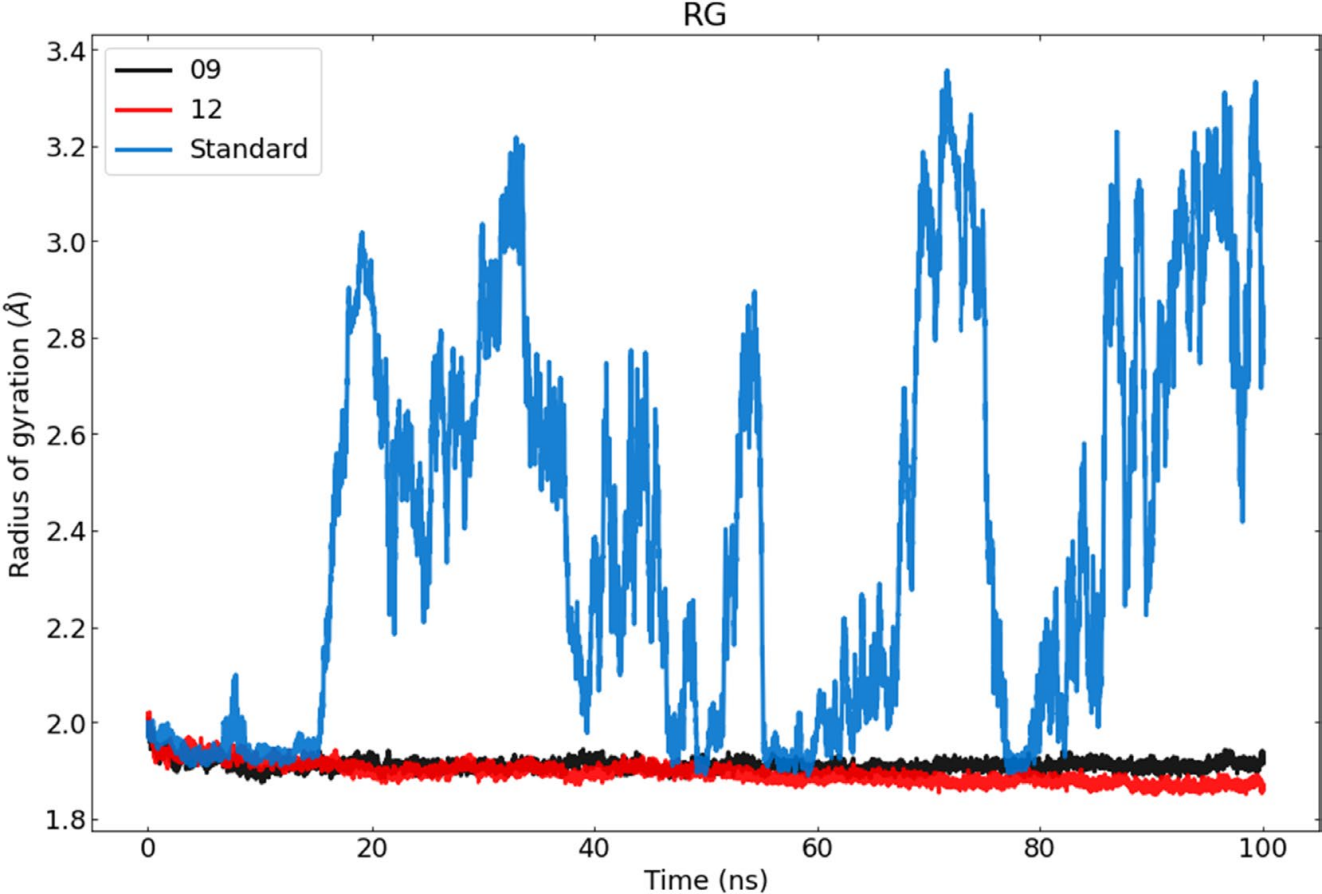
Rg of complex 09 is depicted in black, complex 12 is shown in red, and the standard Rg is depicted in blue.

Throughout the simulation, the Rg parameter measures the compactness of the structure; an increase in Rg indicates that the protein becomes less compact, reflecting greater flexibility and reduced stability [87, 88]. In the present study, Rg was used to evaluate variations in the compactness of the protein–ligand complexes (Figure 10). The data show that the standard complex displays the highest Rg values, suggesting it is more unfolded and less stable than the other complexes. For the standard complex, pronounced fluctuations were observed over the course of the simulation, with Rg peaks at 60 and 80 ns.

Reticuline and calamenene exhibited overall Rg averages of 1.69 Å and 1.73 Å, respectively. Their Rg trajectories over the 100 ns simulation revealed remarkably similar behaviour. During the initial phase (up to ≈20 ns), both complexes showed an upward trend in Rg, with complex 12 (calamenene) presenting the larger increase. Thereafter, neither complex displayed notable changes, maintaining stability and compactness until the end of the simulation.

#### 3.4.4 Hydrogen Bond Analysis

**FIGURE 11:**
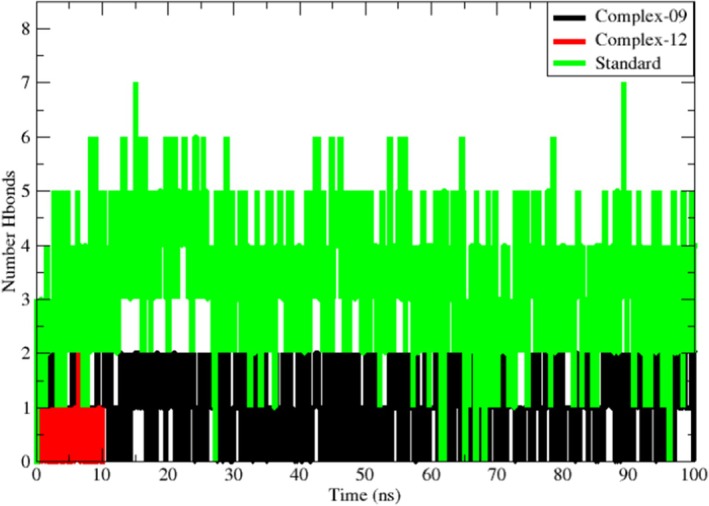
Number of hydrogen bonds that contribute to the stability of the complexes (09 and standard).

The stability of each complex was also examined through hydrogen bonds (H‐bonds). Geometric analysis of these bonds is crucial for understanding biomolecular interactions, as H‐bonds play a key role in preserving structural integrity. During molecular‐dynamics simulations, the formation and persistence of H‐bonds are essential for maintaining complex stability [89]. Figure 11 presents the hydrogen‐bond profiles, showing the number of H‐bonds formed between the protein and each ligand (09, 12 and standard). Hydrogen‐bond interactions, their lifetimes and relative abundances were quantified with VMD and are summarised in Table 8 [90, 91].

For complex 09, the stability of the system during the simulation was upheld mainly through contacts with residues GLY144 and HIS221; reticuline did not reproduce any of the H‐bonds observed in the docking pose. Overall, complex 09 displayed few H‐bonds, most of which occurred between 30 ns and 60 ns. Complex 12 has the fewest hydrogen bonds and does not retain any of the H‐bonds observed in the docking position. In contrast, both H‐bonds present in the docked pose of the standard complex were retained, and additional H‐bonds formed during the trajectory. The stability of this complex was maintained through interactions with residues GLY144, GLY94 and ALA191. The bond with GLY144 showed the highest occupancy (75.05%), whereas GLY94 and ALA191 displayed occupancies of 59.28% and 57.58%, respectively. If the hydrogen bond occupancy rate exceeds 100%, it means that several atom pairs interact to create hydrogen bonds. Complex 12 contains fewer total hydrogen bonds and a lower hydrogen bond occupancy rate than complex 09. The total number of hydrogen bonds and their occupancy rate affect the stability of each system (Table 7). The number and occupancy rate of hydrogen bonds are important determinants in the protein–ligand complex's interaction stability.

**TABLE 7:** Analysis of hydrogen bond occupancies for each complex throughout the molecular dynamics (MD) simulation.

No	Donor acceptor	Occupancy
Complex‐09	LIG285‐Main‐N GLY144‐Main‐O LIG285‐Side‐O2 HIS221‐Side‐ND1	72.36% 26.45%
Complex‐12	SER34‐Main‐N LIG285‐Side‐O3	0.60%
Standard	LIG285‐Main‐N GLY144‐Main‐O LIG285‐Side‐O8 GLY94‐Main‐O LIG285‐Side‐O6 ALA191‐Main‐O	75.05% 59.28% 57.58%

#### 3.4.5 PCA and DCCM Evaluation

PCA is one of the most valuable parameters and functions in significant roles to ensure the stability of the ligand‐protein complex from MD simulation trajectories through the segregation of global slow motions from local fast motions [92, 93].

PCA has enabled the identification of conformational shifts in protein–ligand complexes and the identification and understanding of key coherent movements across different regions. PCA was employed to identify the most significant molecular movements in each simulation, thereby enhancing the understanding of how protein dynamics influence ligand binding. To achieve this objective, the software packages RStudio and Bio3D were utilised [94]. The results were displayed as eigen fractions, which indicate the proportion of variance. These fractions were derived using a covariance matrix consisting of 20 eigen models.

**FIGURE 12:**
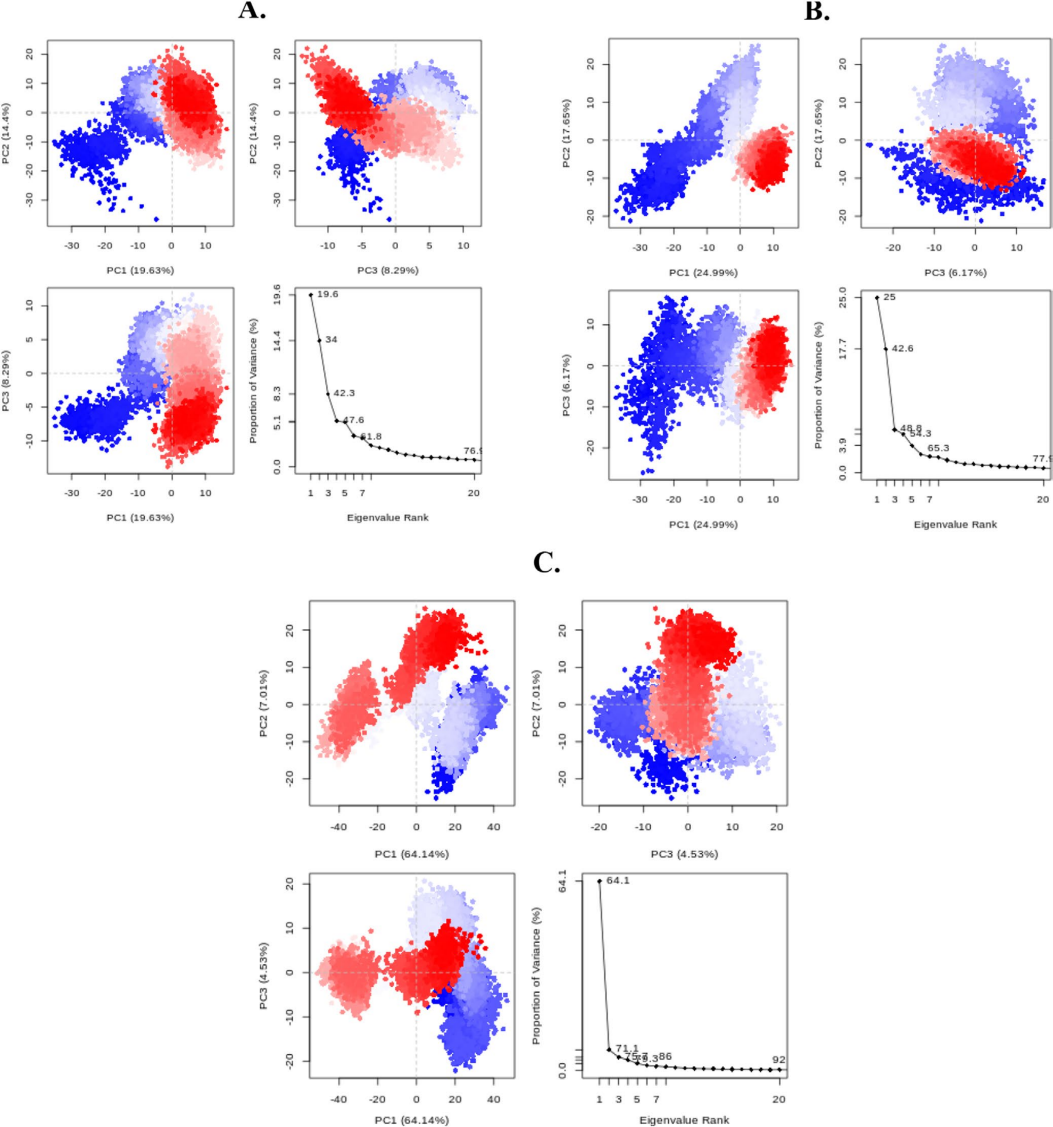
PCA plots of the protein backbone in the complex, comprising graphs of PC2 versus PC1, and an eigenvalue rank plot with the cumulative variance annotated for each data point. (A) Reticuline, (B) calamenene, (C) epirubicin.

Figure 12 shows that backbone mobility in the MD trajectories is dominated by the first three principal components (PCs). In the figure, blue denotes the greatest motions, white indicates moderate movements and red reflects the least flexible regions.

The distribution of the 20 leading PCs for the reticuline, calamenene and epirubicin systems accounts for 76.1%, 77.9% and 92% of total variance, respectively. The reticuline system displays a narrower range of possibilities and lower flexibility than the epirubicin and calamenene systems. For reticuline, the first three eigenvectors explain 19.63%, 19.63% and 8.29% of the protein's variance (Figure 12A); for calamenene, the corresponding values are 24.99%, 24.99% and 6.17% (Figure 12B); and for epirubicin, they are 64.14%, 64.14% and 4.53% (Figure 12C). The high PC1 value for epirubicin (64.14%) suggests that this complex undergoes the largest conformational change.

**FIGURE 13:**
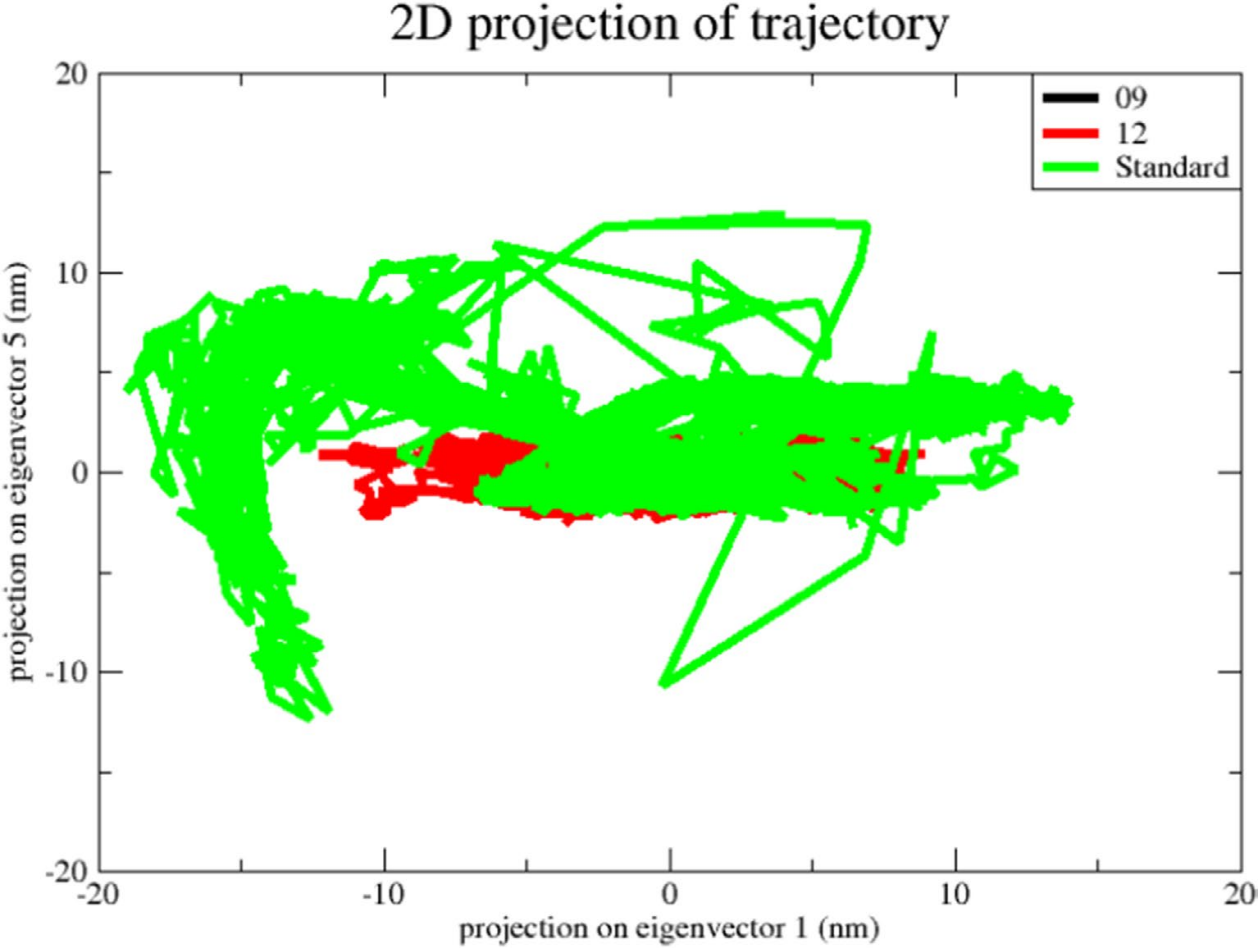
Projection of Cα atoms in essential subspace along the first two eigenvectors of reticuline (black), calamenene (red) and epirubicin (green).

To explore ligand–protein dynamics further, a two‐dimensional projection based on PC1 and PC2 was generated. Figure 13 illustrates the distribution of conformations for the reticuline, calamenene and epirubicin complexes within this critical subspace. A compact cluster indicates a stable complex confined to a reduced phase space, whereas a broad distribution denotes a less stable complex occupying an expanded phase space. The reticuline complex is confined to a more restricted phase space, while calamenene and epirubicin sample significantly larger regions, indicating that the reticuline system is the most stable of the three.

**FIGURE 14:**
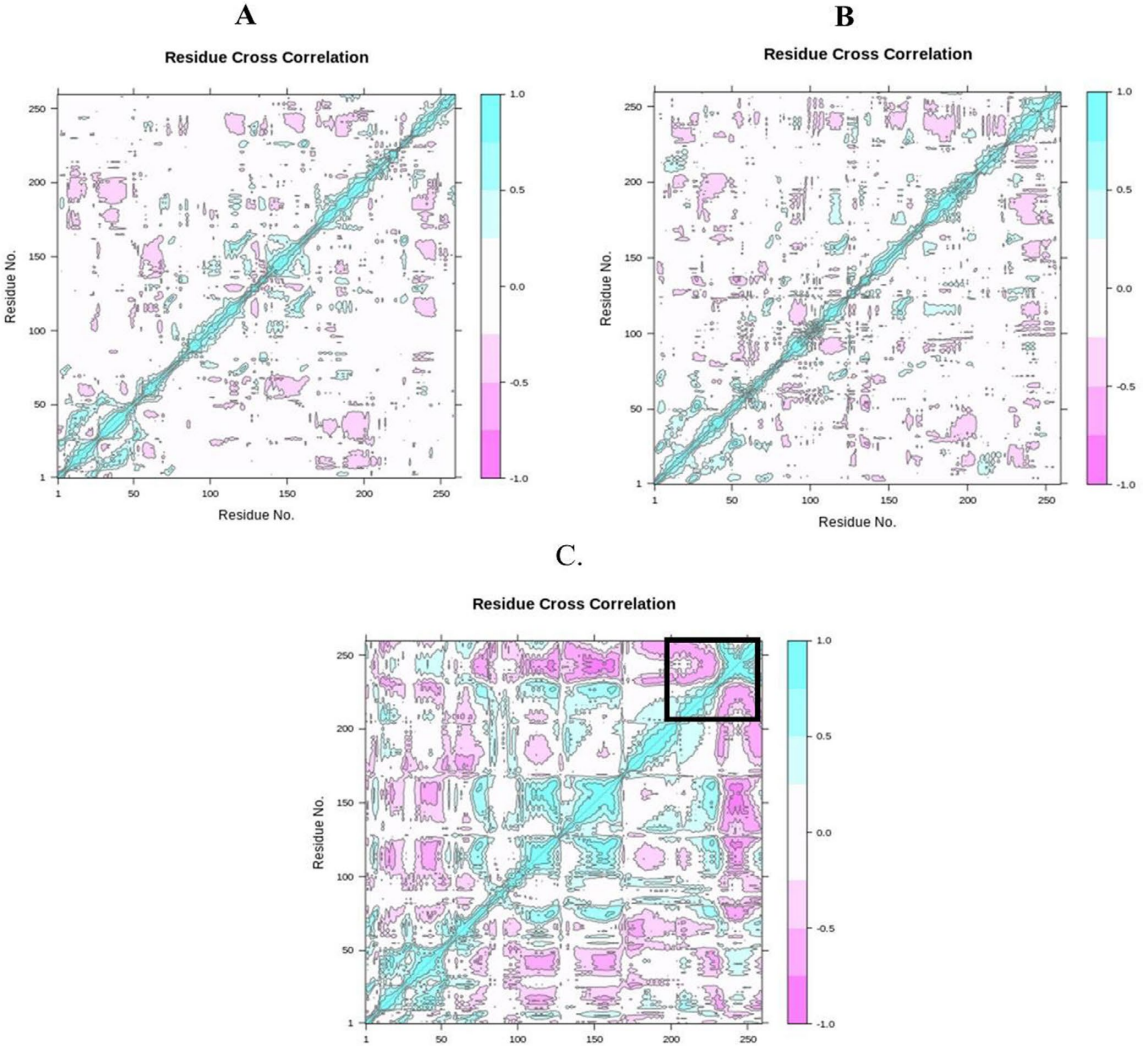
Cα‐residues cross‐correlation profiles for the protein–ligand complexes (A) reticuline, (B) calamenene, (C) epirubicin.

Conformational changes in the target protein were also probed with a DCCM analysis of all Cα atoms. The resulting two‐dimensional diagrams (Figure 14) depict correlated residue motions over the simulation: darker colours represent stronger correlations, with values near 1 indicating residues that move together and values near −1 indicating residues that move in opposite directions [95].

Comparison of the DCCMs shows that the correlation patterns of complexes 09 (reticuline) and 12 (calamenene) differ markedly from those of the standard system. Between reticuline and calamenene, there is no notable difference in positively correlated motions; however, the reticuline system exhibits a pronounced reduction in negatively correlated motions (highlighted by dashed boxes in Figure 14A,B), suggesting that the protein attains a more stable conformational state after reticuline binding. By contrast, the standard system shows substantial increases in both positive and negative correlations, indicating extensive changes in protein dynamics and a tendency toward a more compact conformation following epirubicin binding.

#### 3.4.6 Binding Free Energy Analysis

To assess the molecular interactions within the protein–ligand complexes, binding free energy (ΔG) was calculated with the MM‐PBSA approach, which considers both bonded and non‐bonded contributions. Table 8 summarises the energy components used in this calculation: van der Waals interactions (ΔEVDW), electrostatic interactions (ΔEEEL), the polar‐solvation term (ΔGPB), the non‐polar‐solvation term (ΔGNP), dispersion forces (ΔGDISP) and the overall binding energy (ΔG) [R].

**TABLE 8:** Outcomes of the binding free‐energy.

No	ΔEVDW (kJ/mol)	ΔEEEL (kJ/mol)	ΔGPB (kJ/mol)	ΔGNP (kJ/mol)	ΔGDISP (kJ/mol)	ΔG binding (kJ/mol)
Reticuline (09)	−42.04	−138.20	157.41	−4.15	0.00	−26.99
Calamenene (12)	−10.19	−0.86	4.05	−1.55	0.00	−8.56
Epirubicin (standard)	−53.93	−170.59	193.96	−5.52	0.00	−36.08

The binding‐free‐energy profiles for reticuline, calamenene and the standard deviate from the docking results, a difference that can be attributed to the simplifications inherent in docking—rigid receptors, approximate scoring functions and limited conformational sampling—compared with the more comprehensive, dynamic representation afforded by MD simulations.

Although the MM‐PBSA binding free energy of compound 09 (reticuline, −26.99 kJ/mol) is slightly less favourable than that of the standard epirubicin (−36.08 kJ/mol), it remains relatively close to the standard. Importantly, compound 09 exhibited a better molecular docking score (−8.4 kcal/mol) compared to the standard (−5.7 kcal/mol), suggesting a stronger initial binding affinity at the active site of 17β‐HSD1.

Additionally, reticuline demonstrated greater structural stability during the 100 ns molecular dynamics simulation, as evidenced by lower RMSD and RMSF values, a more compact Rg, consistent hydrogen bond formation, and favourable PCA/DCCM profiles. These parameters collectively support the notion that reticuline forms a stable and specific interaction with the target protein over time.

Furthermore, reticuline showed favourable ADMET properties, including good Caco‐2 permeability, acceptable hERG liability, and no predicted hepatotoxicity or carcinogenicity, making it a potentially safer alternative to the cytotoxic standard drug.

Based on this comprehensive evaluation—including docking, dynamics, free energy decomposition and pharmacokinetic profiling—we consider compound 09 a promising lead scaffold, despite the slightly higher ΔG binding value.

**FIGURE 15:**
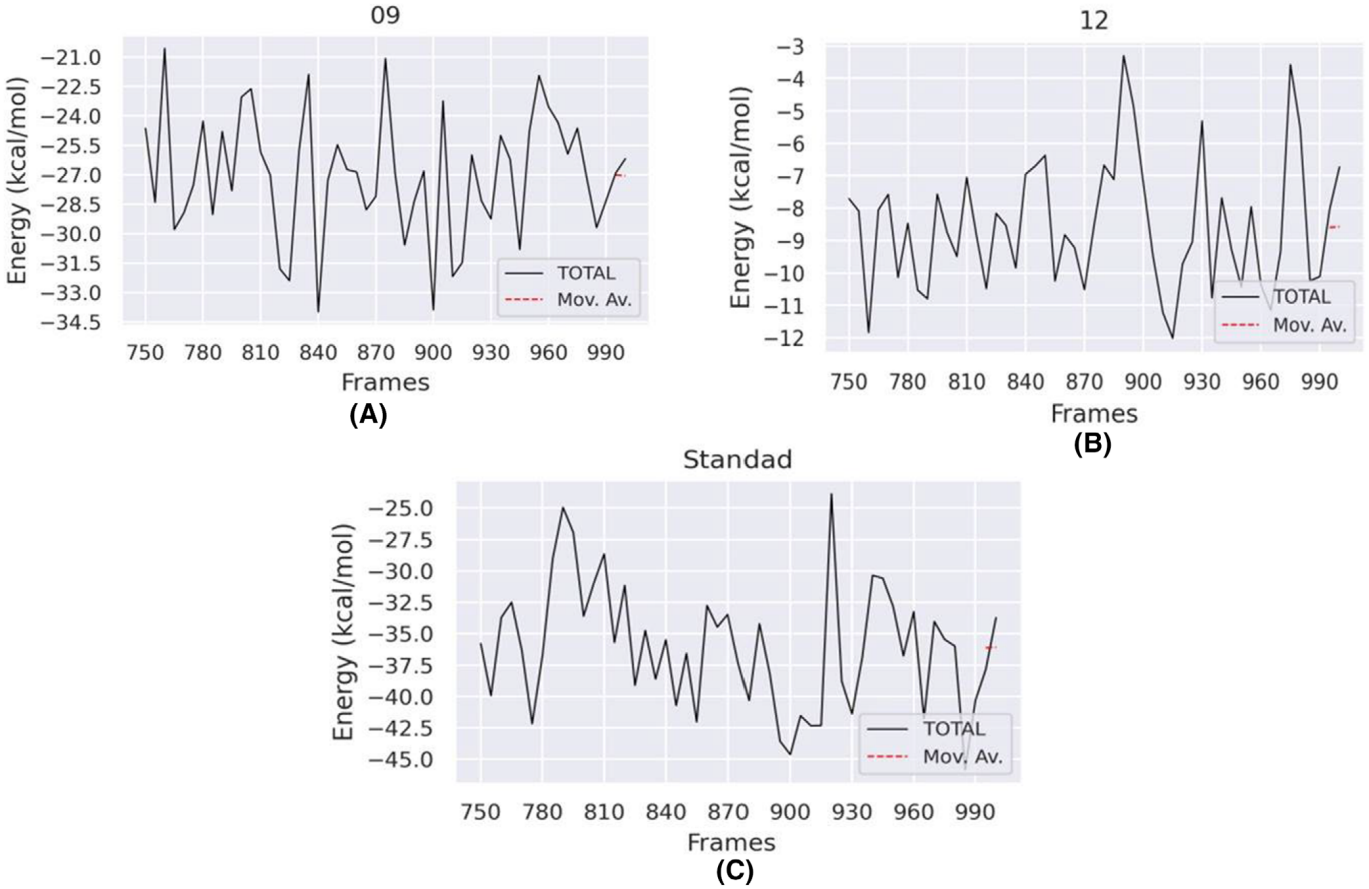
Graphically displayed the binding free energy for reticuline (a), calamenene (b) and epirubicin (c).

Consistent with the docking data, the compounds present comparable free‐binding energies overall. Epirubicin yields a higher binding‐energy value than reticuline, whereas the calamenene complex shows a lower binding energy, indicating a weaker interaction between calamenene and the active site of the target protein (Figure 15 and Table 8).

## 4 Conclusions

This study successfully identified two bioactive compounds (reticuline and calamenene) isolated from *Annona muricata* as promising inhibitors of 17β‐hydroxysteroid dehydrogenase type 1 (17β‐HSD1). Molecular‐docking studies revealed notable binding affinities for both ligands at the enzyme's active site, suggesting their capacity to disrupt catalytic function. ADMET analysis further indicated favourable pharmacokinetic profiles: Reticuline met all Lipinski criteria, showed Caco‐2 permeability of 0.919, acted solely as a non‐inhibitory P‐gp substrate, was predicted to be a selective CYP3A4 substrate with a prolonged plasma half‐life, and triggered no DILI or carcinogenic alerts—only moderate hERG/neuro warnings. Calamenene breached Lipinski only for log P; nevertheless, it retained comparable permeability, functioned as a P‐gp inhibitor, underwent the same CYP3A4‐centred metabolism with a reasonable half‐life, and presented no DILI or genotoxicity warnings, with moderate hERG risk. Molecular‐dynamics simulations corroborated the stability of both ligands within the binding pocket, demonstrating structural integrity and consistent performance under biologically realistic conditions.

Although our integrated in silico strategy offers rapid insights, it is necessarily grounded in approximations. Consequently, the predicted affinities and safety profiles of these phytocompounds remain hypotheses and must be verified through enzymatic assays, cell‐based studies and full pharmacokinetic evaluations, both in vitro and in vivo, before translational application.

These results highlight reticuline and calamenene as promising 17β‐HSD1 inhibitors that merit further experimental validation for potential use in breast cancer therapy.

## Author Contributions

**Emad Rashad Sindi:** conceptualization (equal), data curation (equal), methodology (lead), validation (equal), writing – original draft (lead). **Md Jannatul Islam Polash:** conceptualization (lead), investigation (equal), methodology (lead), project administration (equal), writing – original draft (lead). **Guilherme Bastos Alves:** data curation (equal), investigation (equal), methodology (equal), resources (equal), software (equal). **Imren Bayil:** conceptualization (equal), data curation (equal), methodology (equal), resources (equal), software (equal), writing – original draft (equal). **Samson Olusegun Afolabi:** data curation (equal), investigation (equal), project administration (equal), validation (equal), visualization (equal), writing – review and editing (equal). **Hanan M. Alharbi:** data curation (equal), formal analysis (equal), investigation (equal), project administration (equal), resources (equal), software (equal), visualization (equal). **Alaa A. Khojah:** data curation (equal), formal analysis (equal), investigation (equal), project administration (equal), software (equal), writing – review and editing (equal). **Jonas Ivan Nobre Oliveira:** resources (equal), software (equal), supervision (equal), validation (equal), visualization (equal), writing – review and editing (equal). **Aamal A. Al‐Mutairi:** investigation (equal), project administration (equal), software (equal), visualization (equal), writing – review and editing (equal). **Magdi E. A. Zaki:** conceptualization (equal), software (equal), supervision (equal), validation (equal), visualization (equal), writing – review and editing (equal).

## Ethics Statement

The authors have nothing to report.

## Conflicts of Interest

The authors declare no conflicts of interest.

## Data Availability

All data included in manuscript.
